# NtNAC053, A Novel NAC Transcription Factor, Confers Drought and Salt Tolerances in Tobacco

**DOI:** 10.3389/fpls.2022.817106

**Published:** 2022-05-04

**Authors:** Xiaoxu Li, Qi Wang, Cun Guo, Jinhao Sun, Zhiyuan Li, Yaofu Wang, Aiguo Yang, Wenxuan Pu, Yongfeng Guo, Junping Gao, Liuying Wen

**Affiliations:** ^1^Technology Center, China Tobacco Hunan Industrial Co., Ltd., Changsha, China; ^2^Key Laboratory for Tobacco Gene Resources, Tobacco Research Institute, Chinese Academy of Agricultural Sciences, Qingdao, China; ^3^Shandong Peanut Research Institute, Qingdao, China; ^4^Graduate School of Chinese Academy of Agricultural Sciences, Beijing, China

**Keywords:** tobacco, NtNAC053, ABA, abiotic stress, antioxidant system

## Abstract

The NAC (NAM, ATAF1/2, and CUC2) family acts as one of the largest families of the transcription factor in the plant kingdom and was revealed to function as the important regulators in various environmental stresses. However, a few studies were reported about the biofunctions of the NAC transcription factor in tobacco. In the current study, we characterized a novel NAC transcription factor encoding the gene *NtNAC053* in tobacco, which was significantly up-regulated when exposed to salt and drought treatments. The results of *cis*-acting elements analysis suggested that the promoter region of *NtNAC053* gene possesses a number of stress-responsive elements, and this gene could be induced by exogenous abscisic acid (ABA) treatment. Moreover, the NtNAC053–GFP fusion protein was localized in the cell nucleus and possessed a transactivation domain in its C-terminal, implying that NtNAC053 may undertake as a transcriptional activator in tobacco. Notably, the overexpression of *NtNAC053* in tobacco resulted in hypersensitivity to ABA treatment. Furthermore, these overexpression lines showed significantly enhanced tolerances to drought and salt stresses. Under salt and drought stresses, these overexpression lines possessed higher superoxide dismutase (SOD), catalase (CAT), and peroxidase (POD) activities. Interestingly, the expressions of putative stress-related genes, including *NtCOR15A*, *NtRAB18*, *NtDREB1A*, *NtERF5*, *NtKAT2*, and *NtERD11*, were up-regulated in these overexpression lines when subjected to salt and drought stresses. The clues provided in our study suggested that the *NtNAC053* gene encodes a novel NAC transcription factor and could confer the drought and salt stress tolerances by inspiring the downstream stress-responsive genes and antioxidant system in tobacco.

## Introduction

Transcription factors play a pivotal role in regulating the expression of target genes involved in plant growth, development, and response to environmental stresses (Qu and Zhu, 2006). The NAC protein family has been reported to be one of the largest plant-specific transcription factor families. Its name is based on three transcription factors: no apical meristem (NAM), *Arabidopsis* transcription activation factor (ATAF1,2), and cup-shaped cotyledon (CUC) (Souer et al., 1996; Aida et al., 1997). NAC transcription factors harbor a highly conserved DNA-binding domain at the N-terminus and a variable transcription regulation region (TRR) at the C-terminus (Ernst et al., 2004; Olsen et al., 2005). Generally, the N-terminal DNA-binding domain of NAC protein possesses approximately 150 amino acid residues divided into five subdomains (A-E), which are related to the formation of dimer, functional diversity, and DNA-binding activity (Olsen et al., 2005). The C-terminal TRR of NAC protein functions in conferring regulation diversity of transcriptional activation activity (Yamaguchi et al., 2010; Puranik et al., 2011). Furthermore, the transmembrane domain is also found in several NAC proteins, which function in plasma membrane anchoring (Kim et al., 2010).

The *NAC* gene was first identified from petunias, in which it was involved in the shoot apical meristems development and primordium formation (Souer et al., 1996). In the plant kingdom, the NAC proteins have been reported to play important roles in various biological processes, including lateral root development (Xie et al., 2000; He et al., 2005; Zhang et al., 2018), vascular development (Zhao et al., 2016), development of shoot apical meristem (Takada et al., 2001; Larsson et al., 2012), the formation of the adventitious shoot (Hibara et al., 2003), seed germination (Park et al., 2011), flower formation (Hendelman et al., 2013), secondary wall synthesis (Mitsuda et al., 2007; Zhao et al., 2010), and leaf senescence (Guo and Gan, 2006). So far, the NAC family has been identified in a large number of plants, including *Arabidopsis* (105) (Ooka et al., 2003), tobacco (*Nicotiana tabacum*) (154) (Li et al., 2018), potato (*Solanum tuberosum*) (110) (Singh et al., 2013), white pear (*Pyrus bretschneideri*) (183) (Gong et al., 2019), maize (*Zea mays*) (152) (Shiriga et al., 2014), rice (*Oryza sativa*) (151) (Nuruzzaman et al., 2010), and peanut (*Arachis hypogaea*) (132) (Li P. et al., 2021).

Furthermore, several NAC members have been reported to participate in response to abiotic stresses in *Arabidopsis* and other species. Stress-inducible NAC transcription factors ANAC019, ANAC055, and ANAC072/RD26 were found to improve the drought tolerance *via* regulating the expression of the *ERD1* gene (Tran et al., 2004). Furthermore, *ANAC072* was identified to be involved in the ABA-dependent signaling pathway (Fujita et al., 2004). Notably, the expression of *ANAC002*/*ATAF1* was induced by drought and ABA treatments. Moreover, the *ataf1* knockout mutants displayed a lower survival ratio than wild-type plants under drought conditions, indicating that ATAF1 functions as a regulator in drought stress tolerance (Wu et al., 2009). The *ANAC062*/*NTL6* was reported to play an important role in inducing a drought-resistance response and the overexpression of *NTL6* plants exhibit enhanced resistance to water-deficit conditions (Kim et al., 2012). In addition, the loss of *ANAC035*/*LOV1* function results in hypersensitivity to cold temperature, whereas overexpression of this gene could enhance the cold tolerance (Yoo et al., 2007). In rice, the expression of *OsNAC5* gene could be significantly induced by various stresses, including drought, salt, cold, ABA, and Me-JA. Whereas the overexpression of *OsNAC5* resulted in the improvement of salt stress tolerance (Takasaki et al., 2010). Moreover, the ONAC066 was reported to promote the tolerances against drought and oxidative stresses by activating the transcription of *OsDREB2A* in transgenic rice (Yuan et al., 2019). In wheat, the overexpression of *TaNAC47* in Arabidopsis increases the tolerance to drought stress and sensitivity to ABA (Zhang et al., 2015). Besides, the overexpression of *TaNAC2a* improved drought tolerance in transgenic tobacco (Tang et al., 2012).

Tobacco is one of the most widely cultivated crops worldwide and serviced as a model plant that possesses various primary and secondary metabolisms. However, tobacco has been at a continuous risk from multiple environmental stresses, affecting both yield and quality. In our previous study, the systematic identification of the NAC family was performed in tobacco (Li et al., 2018). However, a few studies were reported about the biofunctions of the NAC transcription factor in tobacco. Notably, in our previous study, the expression of *StNAC053* was significantly up-regulated after salt and drought treatments. The overexpression of this potato gene could enhance the salt and drought tolerance in transgenic *Arabidopsis* (Wang et al., 2021). Considering the close genetic distance of potato and tobacco, the homolog of StNAC053 might also confer salt and drought tolerances in tobacco. To verify this hypothesis, we cloned and characterized an NAC transcription factor, NtNAC053, from tobacco, and systematic analysis was performed to explore the function of this gene in salt and drought stress responses.

## Materials and Methods

### Plant Materials and Treatments

Cultivated tobacco K326 was used to analyze the expression pattern of *NtNAC053* in the current study. The root, root tip, stem, stem tip, young leaf, and senescent leaf were collected. For salt, drought, and ABA treatments, tobacco seedlings were transferred to MS liquid medium supplemented with 100 mM NaCl, 300 mM mannitol, and 10 μM ABA for 0, 1, 3, and 6 h, respectively. For cold treatment, tobacco seedlings were treated at 4°C for 0, 1, 3, and 6 h. All of the harvested samples were stored at –80°C before RNA extraction was performed. Three biological replicates were used for each sample.

### RNA Extraction and qPCR Analysis

Ultrapure RNA Kit (cwbiotech, Beijing, China) was used to extract total RNA, then the first cDNA strand was synthesized using Evo M-MLV Mix Kit with gDNA Clean for qPCR (Accurate Biotechnology, Changsha, China). Expression of stress-responsive genes (*NtDREB1A*, *NtERF5*, *NtKAT2*, *NtCOR15A*, *NtRAB18*, *NtERD11*, *NtNHX1*, and *NtSOS1*) and ABA signaling genes from tobacco was detected. The qRT-PCR reactions were carried out in Roche LightCycler 480 Real-Time PCR instrument with SYBR Green Premix Pro Taq HS qPCR Kit (Accurate Biotechnology, Changsha, China). The tobacco ribosomal protein gene *L25* (GenBank No. L18908) was used as a control (Li Z. et al., 2021). All experimental data were obtained through three technical repetitions, and the relative expression level was calculated by 2^–ΔΔCT^ method (Livak and Schmittgen, 2001). The details of the primers in this study are provided in Supplementary Table 1.

### Cloning and Sequence Analysis of *NtNAC053*

BLASTP search with an *E*-value cutoff of 0.0001 was carried out in SGN^1^ using potato StNAC053 protein sequence as a query. The candidate gene (Nitab4.5_0000322g0110.1) was obtained and then named as *NtNAC053*. The CDS of *NtNAC053* was amplified by RT-PCR. The purified PCR products were subcloned into the pEASY-Blunt vector, and the constructed vector was then transformed into *Escherichia coli* 5α competent cells (Trans Gen, Beijing, China). After sequencing, the recombinant plasmid was named *Blunt-NtNAC053*. The physicochemical properties of the NtNAC053 protein, including molecular weight (MW) and isoelectric point (pI), were predicated using the ProtParam online tool^2^. The nuclear localization signal (NLS) of NAC proteins was predicted *via* cNLS Mapper^3^. The DNAMAN software (Lynnon Biosoft) was used to perform multiple sequence alignment. A neighbor-joining tree was generated using MEGA 6 with 1,000 replications. The NAC protein sequences were obtained from the TAIR^4^, SGN (see text footnote 1), and NCBI. The conserved motifs of NAC protein sequences were analyzed *via* MEME^5^ with default parameters. The collinearity relationship between NtNAC053 and StNAC053 was analyzed using TBtools (Chen et al., 2020).

To assess the promoter *cis*-acting elements of the *NtNAC053* gene, the sequences 2,000 bp upstream of the *NtNAC053* gene were extracted from the genome database. The obtained sequences were subjected to PlantCARE^6^ platform analysis to further search for the putative *cis*-elements in their promoter regions.

### Subcellular Localization and Transactivation Assay

The CDS (without stop codon) of *NtNAC053* gene was amplified from *Blunt*-*NtNAC053* using PCR. Then the purified PCR products was inserted into the PYG57 vector, which started by the CaMV-35S promoter and contained the GFP fragment. The construct was transformed into *Agrobacterium* competent cell GV3101, and transiently expressed in the leaves of *Nicotiana benthamiana*. Simultaneously, the empty vector injected leaves as a control. Three days after the injection, the leaves were soaked in a DAPI staining solution to determine the location of the nucleus. The confocal laser microscope (Leica SP8) was used to observe the fluorescence signal of the fusion protein.

The CDS of *NtNAC053* was amplified and inserted into the *Eco*RI site of the pBridge vector, using an In-fusion HD Cloning Kit (Takara, Shiga, Japan) to fuse with a GAL4 DNA binding domain. The constructs and the control vector were introduced into the yeast strain AH109 separately, followed by growing yeasts on SD/-Trp, and SD/-Trp supplemented with X-Gal for 4 days at 30°C. The transcriptional activation activities were evaluated based on the growth status of different transformants.

### Generation of *NtNAC053* Transgenic Tobacco Plants

The coding sequence of *NtNAC053* gene was amplified from the cDNA and inserted into the pCHF3 vector, which was driven by the CaMV-35S promoter, to complete the construction of the overexpression vector. The pCHF3 plasmid containing the *NtNAC053* gene was transformed into tobacco leaves by the *Agrobacterium*-mediated method (Buschmann, 2016). T0 transgenic seeds were screened on MS medium containing 50 mg/L kanamycin to obtain *NtNAC053* overexpression plants. T3 generation homozygous seedlings were used for further experiments.

### Seedling Growth Assays

For seed germination assays, T3 generation seeds and wild-type (K326) seeds were sterilized and evenly sown on MS medium containing 1 μM ABA, 100 mM NaCl, and 150 mM NaCl, respectively. After cultivation for 14 days in a growth chamber (25°C, continuous light), the germination rates were calculated with three replications.

For root elongation assays, tobacco seedlings were grown in vertical MS medium for 14 days, then transferred to MS medium with NaCl (100 and 150 mM) and ABA (1 μM), respectively. The length of the primary root was measured after 14 days with three replications.

### Drought Stress Tolerance Assays of *NtNAC053* Transgenic Tobacco Plants

For the drought tolerance assays, T3 transgenic and wild-type (K326) tobacco seeds were sown into the soil mixture evenly and kept in normal conditions. Six weeks later, the tobacco seedlings were treated by drought stress for 10 days. Then the seedlings were re-watered for 3 days, and the survival rates of tobacco seedlings were calculated. The leaves of the seedlings were collected and air-dried for 3 h, then leaf weight was recorded at five time points (0, 45, 90, 135, and 180 min). The rate of water loss was calculated based on the leaves’ weight changes.

### Physiological Measurements

The physiological parameters, including contents of MDA and hydrogen peroxide, and the enzymatic activities of superoxide (SOD), peroxidase (POD), and catalase (CAT) were measured as previously described (He et al., 2019a). Three biological replicates were performed.

### Yeast One Hybrid Assay

For yeast one-hybrid assay, the full-length CDS sequence of *NtNAC053* transcription factor was inserted into pGAD424 to construct a *pGAD424-NtNAC053* recombinant vector. Furthermore, the *pGAD424-NtNAC053* recombinant vector was co-transformed with different *LacZ* reporter gene constructs containing three tandem reporter sequences into yeast strain YM4271. The β-galactosidase activity was assessed according to the manufacturer’s instruction (Clontech, United States).

### Statistical Analysis

The GraphPad Prism 8 (*t*-test) was used to analyze the statistical significance. *P* < 0.05, 0.01, or 0.001 were regarded as significantly different from the control. All data were obtained from three replicates.

## Results

### Isolation and Characterization of the *NtNAC053* Gene

The NAC transcription factors were reported to play multiple roles in response to abiotic stresses. In our previous study, the StNAC053 was found to confer salt and drought tolerance in transgenic *Arabidopsis* (Wang et al., 2021). To identify the homolog of StNAC053 in tobacco, the BLASTP search was carried out in SGN (see text footnote 1) using the StNAC053 protein sequence as a query. The candidate gene (Nitab4.5_0000322g0110.1) was obtained and then named as *NtNAC053*. Furthermore, the *NtNAC053* gene was cloned from cultivated tobacco K326. The full-length coding sequence of *NtNAC053* is 897 bp, which encodes a 298 amino acid protein. The molecular weight (MW) and theoretical isoelectric point (pI) of the protein are 34.247 kDa and 6.56, respectively. Multiple sequence alignments persuaded that NtNAC053 possesses a conserved NAC domain (8-162) in the N-terminal region and a variable domain at the C-terminal region. The NAC domain consists of five subdomains, including subdomain A-E, which have been predicted to function as the DNA-binding domain together. Furthermore, a novel nuclear localization signal (NLS) was identified in the subdomain D (Supplementary Figure 1).

### Phylogenetic and Syntenic Analysis of the NtNAC053

To explore the phylogenetic relationship of NtNAC053, a neighbor-joining (NJ) tree containing the selected novel NAC transcription factor, including seven members from *Arabidopsis*, three from potato, and two from wheat. The results persuaded that NtNAC053 was clustered with StNAC053, and both of these two members fall into the ATAF subfamily (Supplementary Figure 2A). Furthermore, the conserved motifs of NtNAC053 and these selected NAC members were predicted by the MEME tools. As a result, 15 conserved motifs were identified (Supplementary Figure 3). Notably, motif 3, 1, 2, and 4 represented the NAC domain and were shared by all tested members (Supplementary Figure 2B). Besides, it was found that motif 12 may be specific to the dicotyledonous members of ATAF subfamily.

To further study the evolutionary relationship of *NtNAC053* gene, syntenic analysis was performed for tobacco and potato (Supplementary Figure 2C). The result showed that *NtNAC053* was the ortholog gene of *StNAC053*.

### Expression Patterns and Promoter Analysis of *NtNAC053*

**FIGURE 1:**
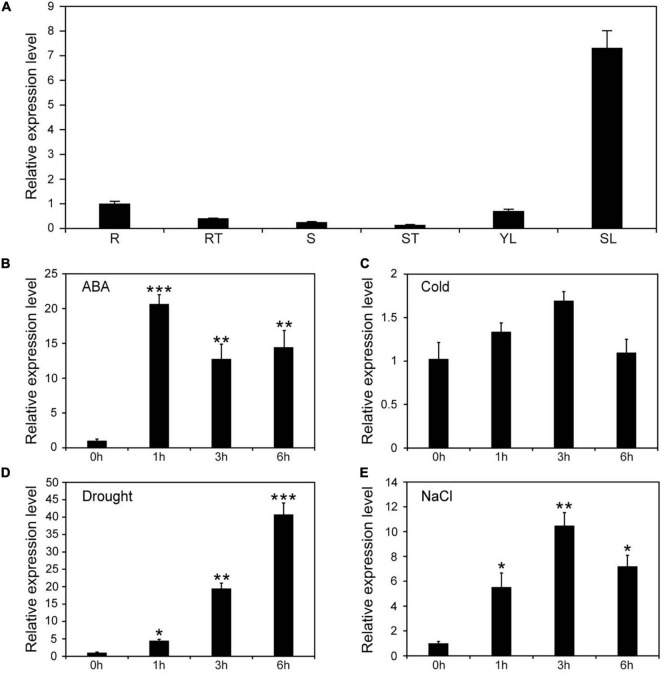
Expression patterns of *NtNAC053* in tobacco. **(A)** The expression pattern of *NtNAC053* in tested tissues. R: root; RT: root tip; S: stem; ST: stem tip; YL: young leaf; SL: senescent leaf; Expression pattern of *NtNAC053* under ABA **(B)**, cold **(C)**, drought **(D)**, and salt **(E)** treatments. The data were means ± SD from three independent replications. * p < 0.05, ** p < 0.01, *** p < 0.001 (*t*-tests).

To explore the potential function of NtNAC053, the *cis*-acting elements and the expression pattern of *NtNAC053* in different tissues were investigated. Interestingly, the *cis*-acting elements analysis revealed that five elements related to ABA responsiveness (ABRE) were identified in the promoter region of the *NtNAC053* gene (Supplementary Figure 4). As shown in Figure 1A, the expression of *NtNAC053* had a higher level in tobacco root and young leaves, and had a lower level in the root tip, stem, and stem tip. Interestingly, the expression of *NtNAC053* had a high level in senescent leaf. Furthermore, we examined the expression pattern of *NtNAC053* under various treatments, including drought, salt, cold, and ABA. For the drought and ABA treatments, the expression levels of *NtNAC053* were up-regulated before the first 6 h, respectively (Figures 1B,D). For the salt treatment, the expression levels of *NtNAC053* were also up-regulated and reached the peak at 3 h (Figure 1E). For the cold treatment, no significant alteration was detected (Figure 1C).

### Subcellular Localization and Transactivation Activity Assay

**FIGURE 2:**
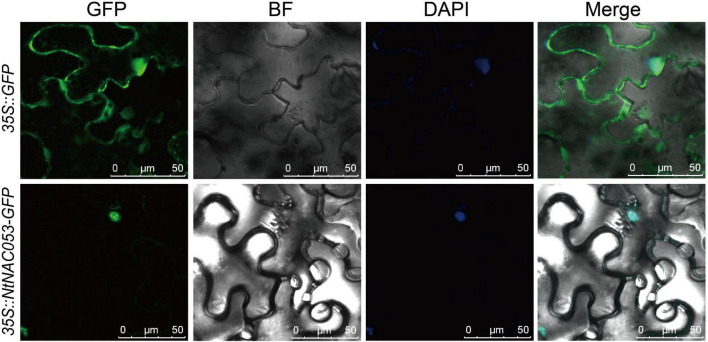
The subcellular localization of the NtNAC053 protein in *Nicotiana benthamiana* epidermal cells. Transient expression of GFP and NtNAC053-GFP in *N. benthamiana* epidermal cells. Bar = 50 μM, DAPI, 4,6-diamidino-2-phenylindole for nuclear staining.

To further identify the function of NtNAC053, the subcellular localization was investigated. Generally, the coding sequence of *NtNAC053* gene was fused with GFP and transiently expressed in the *N. benthamiana* leaves. As a result, the fluorescence signals of 35S:GFP control displayed as spreading throughout the cells (Figure 2). Meanwhile, the green fluorescent signal of the fused *NtNAC053-GFP* was only observed in the nucleus. This result illustrated that NtNAC053 protein was a nuclear-localized protein.

**FIGURE 3:**
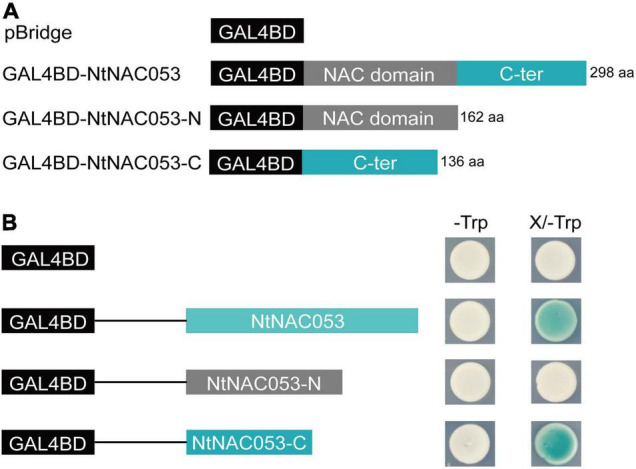
The analysis of transcriptional activation activity of NtNAC053. **(A)** The fusion proteins with the GAL4 DNA-binding domain. **(B)** Transcriptional activation activity assay of NtNAC053 in the yeast strain AH109. The β-galactosidase activity was detected on the SD/-Trp/X-gal medium. NtNAC053-N represented the N-terminal region of NtNAC053 (1-162 aa); NtNAC053-C represented the C-terminal region of NtNAC053 (163-298 aa).

To determine the transcriptional activity of NtNAC053, the full length of *NtNAC053* and two truncated variants, including N-terminal region and C-terminal region, were inserted into the pBridge vector, respectively (Figure 3A). The pBridge empty vector and these three recombinant constructs were then transformed into yeast competent cells. The transformants containing the full length of NtNAC053 and C-terminal region of NtNAC053 were found to show β-galactosidase activity and turn blue on the SD/-Trp/X-gal medium. Whereas the transformants with pBridge empty vector and N-terminal region of NtNAC053 did not show β-galactosidase activity (Figure 3B). Therefore, NtNAC053 could function as a transcriptional activator, and the C-terminal region is critical for this activity.

### The Overexpression of *NtNAC053* in Tobacco Confers Abscisic Acid Hypersensitivity

**FIGURE 4:**
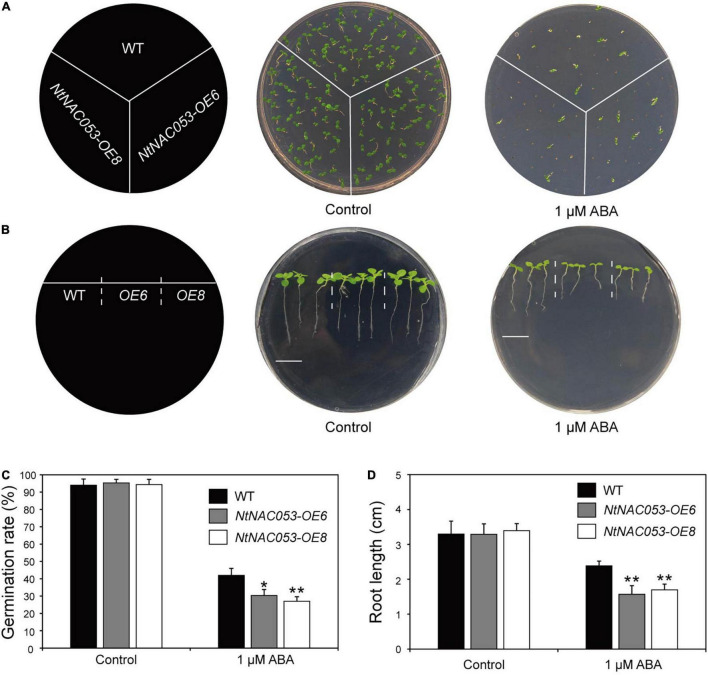
Overexpression of the *NtNAC053* gene confers ABA hypersensitivity. **(A)** Seed germination assay between overexpression lines and wild type under 1 μM ABA treatment. **(B)** Root elongation assay between overexpression lines and wild type under 1 μM ABA treatment. Bar = 1.5 cm. **(C)** The statistics of germination rates under normal conditions and 1 μM ABA treatment. **(D)** The statistics of primary root length under normal conditions and 1 μM ABA treatment. WT, wild type. The data were means ± SD from three independent replications. * p < 0.05, ** p < 0.01 (*t*-tests).

To clarify the potential function of NtNAC053, the transgenic overexpression lines of *NtNAC053* gene were generated in tobacco. The qRT-PCR analysis was used to detect the expression levels of *NtNAC053* gene in these lines. The results revealed the expression levels of this gene in *OE6* and *OE8* reached 47.7- and 61.8-fold than that in wild type (WT) (Supplementary Figure 5). These two lines were then selected to perform phenotyping under different treatments. In our previous survey, the *NtNAC053* gene could be induced by exogenous ABA. Hence, the germination assay and root elongation assay were carried out to assess the ABA sensitivity in the overexpressed lines. As a result, the germination rates seemed to be identical in the germination assay between the overexpressed lines and WT plants. Interestingly, the overexpressed lines had significantly lower germination rates than WT plants when exposed under ABA treatment (Figures 4A,B). Furthermore, the root length seemed to be equivalent in the root elongation assay between the overexpressed lines and WT plants. Notably, the overexpressed lines seemed to be more sensitive than WT plants when treated by exogenous ABA (Figures 4C,D).

### *NtNAC053* Overexpression Enhances Salt Tolerance in Transgenic Tobacco

**FIGURE 5:**
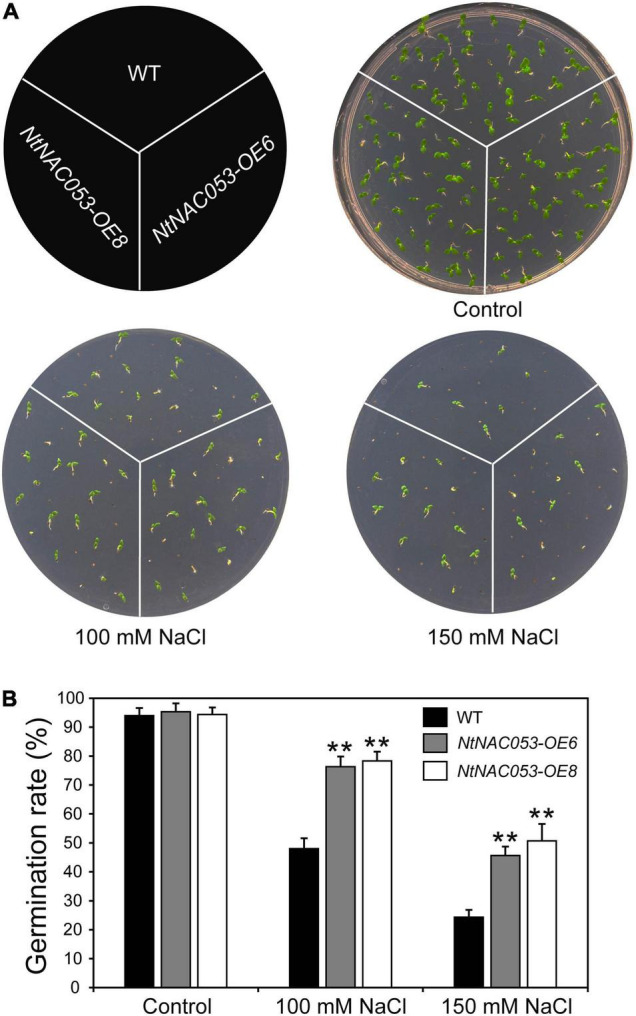
Overexpression of the *NtNAC053* gene promotes seed germination under salt stress treatments. **(A)** Seed germination assay between overexpression lines and wild type under NaCl treatments. **(B)** The statistics of seed germination under normal conditions and NaCl treatments. WT, wild type. The data were means ± SD from three independent replications. ** p < 0.01 (*t*-tests).

To explore whether NtNAC053 regulates the tolerance of tobacco plants to salt stress, we performed a seed germination assay and root elongation assay on MS media with salt treatment. As shown in Figure 5, the seed germination rates of both the overexpression lines and WT were decreased under salt treatment, whereas the overexpression lines displayed higher germination rates than WT seeds under salt treatments.

**FIGURE 6:**
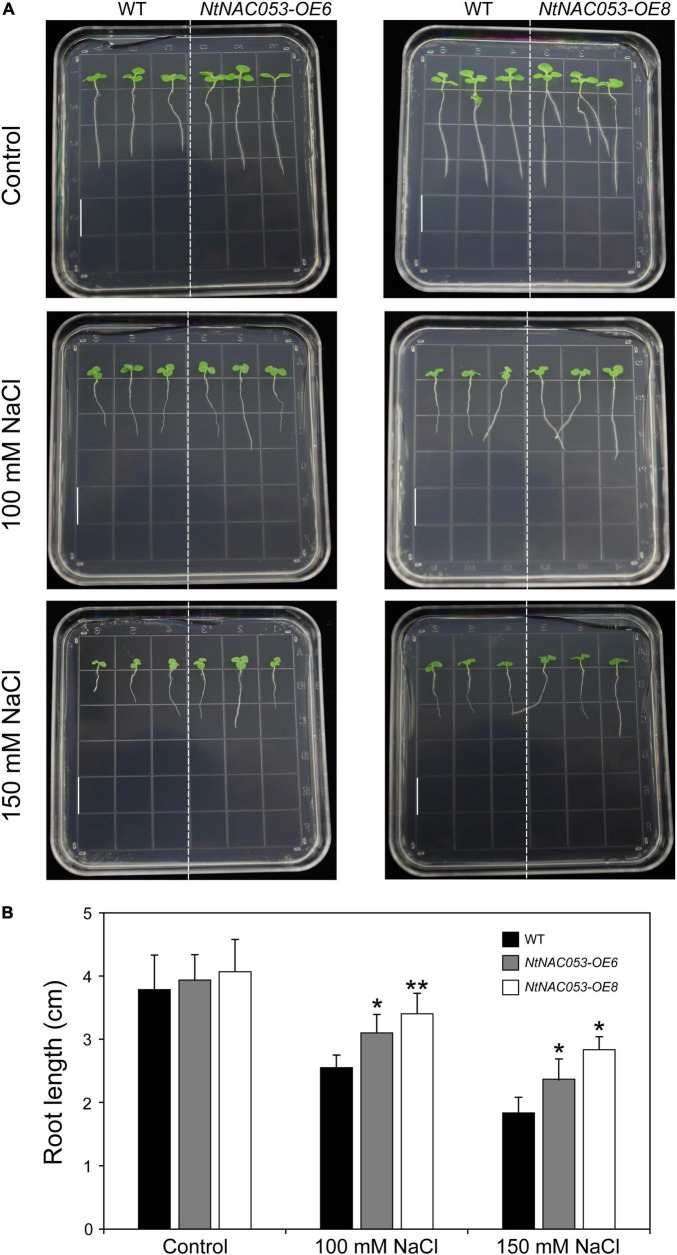
Comparison of root growth between overexpression lines and wild-type tobacco under salt stress treatments. **(A)** The primary root length of overexpression lines and wild-type tobacco under 100 mM and 150 mM NaCl treatments. The primary root length was recorded 7 days after growth. Bar = 1.5 cm. **(B)** The statistics of primary root length under normal conditions and NaCl treatments. WT, wild type. The data were means ± SD from three independent replications. * p < 0.05, ** p < 0.01 (*t*-tests).

As shown in Figure 6, no significant difference was observed in root length between overexpression lines and WT seedlings. However, the primary roots of overexpression seedlings were longer than that of WT on the MS media supplemented with tested concentrations of NaCl.

### *NtNAC053* Overexpression Improves Drought Tolerance in Transgenic Tobacco

**FIGURE 7:**
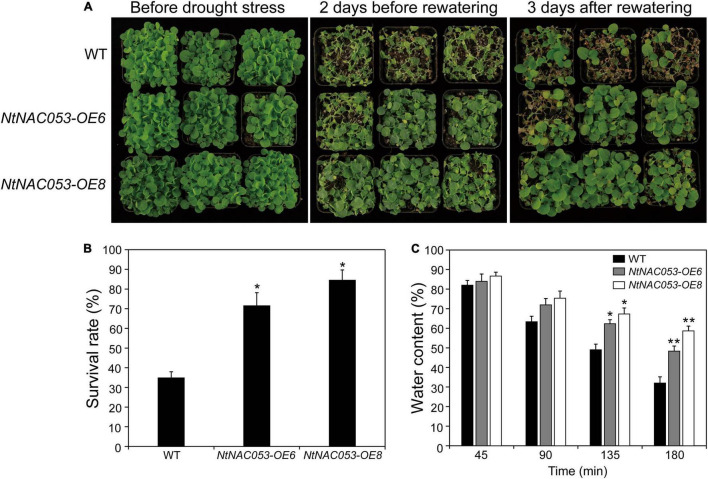
Drought tolerance assay of transgenic *NtNAC053* tobacco. **(A)** Phenotypes of overexpression lines and wild-type tobacco under drought stress. Under normal conditions, the overexpression lines and wild-type tobacco were normally grown for 6 weeks. Overexpression lines displayed higher drought tolerance after 10 days of drought stress and 3 days of recovery. **(B)** The survival rate of the overexpression lines and wild-type tobacco were determined. **(C)** The water content of detached leaves of overexpression lines and wild-type tobacco. WT, wild type. The data were means ± SD from three independent replications. * p < 0.05, ** p < 0.01 (*t*-tests).

To further explore the function of *NtNAC053* gene, the overexpression lines and WT seedlings were treated by the drought stress with no watering for approximately 10 days. The growth of overexpression lines and WT seedlings was almost the same under normal growth conditions. After the drought treatment, overexpression lines and WT seedlings displayed leaf wilting phenotype, whereas WT seedlings were much more extreme than that in overexpression lines (Figure 7A). After 3 days of re-watering, the survival rates of the *OE6* lines (72%) and *OE8* lines (85%) were significantly higher than those of the WT seedlings (35%) (Figure 7B). Additionally, the overexpression lines displayed higher water content than WT seedlings during the 3 h dehydration (Figure 7C). Besides, the drought stress-responsive gene *NtAQP1* was selected to explore the expression in wild type and overexpression lines. Under drought conditions, the expression of *NtAQP1* was significantly higher in overexpression lines (Supplementary Figure 6). These findings suggested that the overexpression of *NtNAC053* gene might enhance the drought tolerance of the tobacco plants.

### *NtNAC053* Overexpression Enhances ROS-Scavenging Capability in Transgenic Tobacco

**FIGURE 8:**
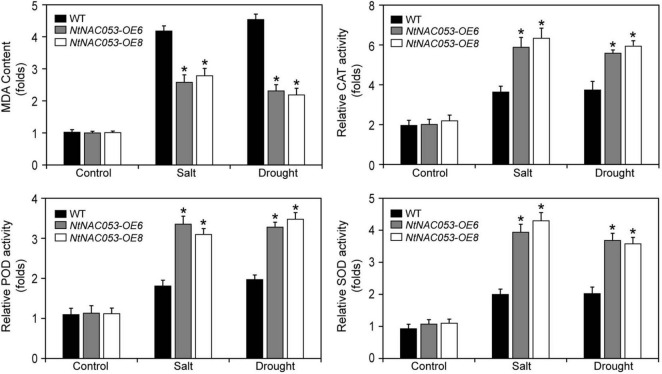
Analysis of the antioxidant enzyme activities and MDA contents in overexpression lines and wild-type tobacco under salt and drought stresses. MDA, malonic dialdehyde; CAT, catalase; POD, peroxidase; SOD; superoxide. WT, wild type. The MDA content and enzyme activity were calculated as folds relative to the level of before treatments. The data were means ± SD from three independent replications. * p < 0.05 (*t*-tests).

To further investigate the function of *NtNAC053*, the stress-related physiological parameters, including catalase (CAT), peroxidase (POD), superoxide (SOD), malonic dialdehyde (MDA), and hydrogen peroxide (H_2_O_2_), were detected (Figure 8 and Supplementary Figure 7). After salt and drought treatments, the content of MDA and H_2_O_2_ in overexpression lines was significantly lower than those in WT plants. However, the activities of CAT, POD, and SOD in overexpression lines were significantly higher than those in WT plants.

### NtNAC053 Regulates the Expression of Stress-Responsive Genes

**FIGURE 9:**
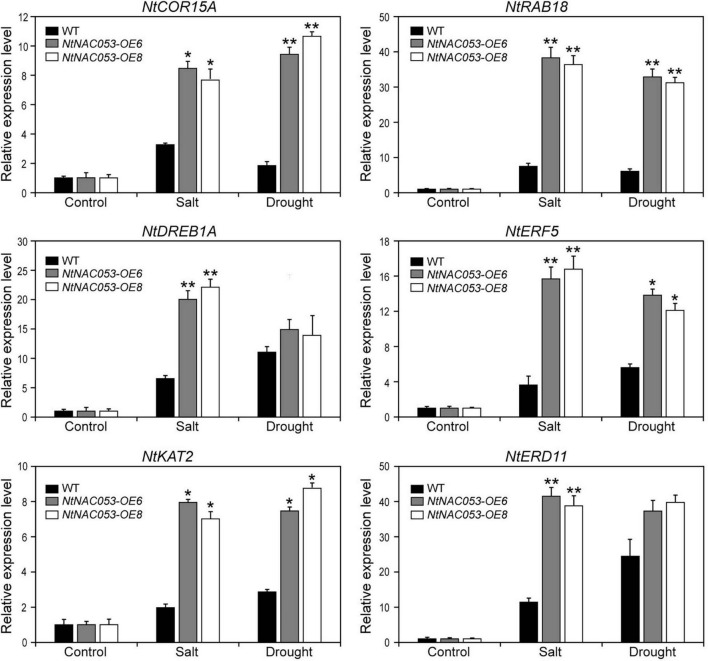
Expression of stress-related genes in overexpression lines and wild-type tobacco under salt and drought stresses. The ratios of gene expression levels were calculated relative to the control, respectively. WT, wild type. The data were means ± SD from three independent replications. * p < 0.05, ** p < 0.01 (*t*-tests).

To further explore the function of *NtNAC053* gene, the expression patterns of stress-responsive genes were detected in overexpression lines and WT plants using qRT-PCR. The expression pattern of the selected stress-responsive genes was calculated as folds relative to the expression level of none treatments. Exposed to salt and drought stresses, the expression levels of several genes were significantly induced in overexpression lines compared to WT plants, including *NtCOR15A*, *NtRAB18*, *NtDREB1A*, *NtERF5*, *NtKAT2*, and *NtERD11* (Figure 9). Besides, we selected two salt stress-responsive genes (*NtNHX1* and *NtSOS1*) and compared their expressions in overexpression lines and WT plants. Under salt conditions, the expressions of *NtNHX1* and *NtSOS1* were significantly higher in overexpression lines (Supplementary Figure 8). Notably, ABA signaling-related genes (*NtPYL6* and *NtSnRK2.4*) were selected for further study. In overexpression lines, these gene expressions were much higher compared to WT plants under drought and salt conditions (Supplementary Figure 9).

### NtNAC053 Binds to NACRS Sequence

**FIGURE 10:**
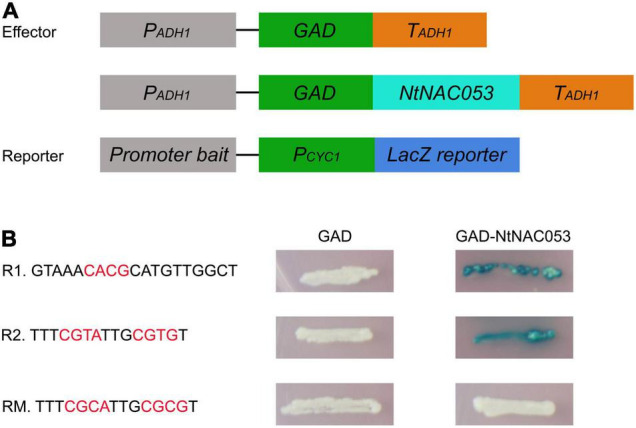
Binding activity of NtNAC053. **(A)** Diagrams illustrating the structure of reporter and effector constructs. **(B)** The sequence of the NACRS (R1 and R2) and mutated NACRS (RM). Different combinations of effector and reporter constructs were co-expressed in yeast strain YM4271.

The NAC transcription factors mainly bind to the NAC recognition sequence (NACRS) with a core sequence of CACG to regulate gene expression, while the reverse complementary sequence is CGT[A/G]. To verify the putative DNA-binding sequences of *NtNAC053*, the NACRS sequence and the reverse complementary sequence were used as reporters (R1 and R2), while the base-substituted fragment was used as the mutant reporter (RM). The activator vector *pGAD-NtNAC053* was co-transformed into the yeast strain with three reporter vectors, respectively. If the NtNAC053 can bind to the tandem sequence of the reporter construct, the GAD will be able to direct *LacZ* expression, resulting in the blue color accumulation. As shown in Figure 10, these yeast one-hybrid experiments confirmed the ability of NtNAC053 to bind to the NACRS sequence *in vivo*.

## Discussion

Plants are suffering from a combination of abiotic and biotic stresses. The abiotic stresses, including salt and drought, affect plant growth and development. In previous studies, several NAC members have been revealed to participate in responses to abiotic stress through ABA signaling (Nakashima et al., 2012; Shao et al., 2015). In the current study, promoter analysis showed that the *NtNAC053* promoter possesses five ABRE motifs, which is an important *cis*-acting element related to ABA signal transduction, indicating this gene might be involved in the ABA signaling pathway (Supplementary Figure 4). To clarify this inference, the expression pattern analysis and overexpression survey were carried out. Interestingly, it was shown that *NtNAC053* was significantly induced by exogenous ABA treatments (Figure 1B). Furthermore, the seed germination rate and root lengths of overexpression lines were significantly lower than those of WT plants, suggesting that overexpression of *NtNAC053* conferred transgenic tobacco plants hypersensitive to ABA (Figure 4). Notably, the expressions of ABA signaling genes, including *NtPYL6* and *NtSnRK2.4*, were much higher in overexpression lines compared to WT plants under salt and drought conditions (Supplementary Figure 9).

It has been reported that NAC transcription factors play critical roles in various abiotic stress responses. However, a few studies were reported about the biofunctions of the NAC transcription factor in tobacco. In our previous work, a tobacco *NAC* gene was found to be dramatically induced by multiple stress treatments (Li et al., 2018), which was identified as the homolog of StNAC053. In our previous study, we found that the overexpression of *StNAC053* gene could enhance the salt and drought tolerance of transgenic *Arabidopsis* (Wang et al., 2021). Considering the close genetic distance of potato and tobacco, the homolog of StNAC053 might confer salt and drought tolerance in tobacco. To verify this hypothesis, a systematic analysis of NtNAC053 was performed. The StNAC053 had been reported to fall into the ATAF subfamily. Hence, several reported ATAF family members from potato, wheat, and Arabidopsis were selected to perform the phylogenetic analysis. Phylogenetic analysis showed that NtNAC053 was clustered with StNAC053 in ATAF subfamily, implying that NtNAC053 served as a homolog of StNAC053. In tobacco, we found that *NtNAC053* was significantly up-regulated when subjected to salt and drought stresses, suggesting that NtNAC053 may function as a regulator in response to salt and drought stresses (Figure 1). Furthermore, the seed germination rate and root lengths of WT plants were significantly lower than those of overexpression lines under salt treatment (Figures 5, 6). Meanwhile, overexpression lines showed higher survival rates compared to WT plants when exposed to drought stress (Figures 7A,B). In addition, the leaves of overexpression lines had a lower water loss rate than those of WT plants under drought conditions (Figure 7C). Considering these results, the overexpression of *NtNAC053* gene could enhance the tolerance to salt and drought stresses in tobacco.

Additionally, massive amounts of ROS were produced when plants were exposed to multiple stresses. Generally, excessive ROS could destroy antioxidant systems and the integrity of cell membrane, resulting in the accumulation of MDA (Moore and Roberts, 1998; Gill and Tuteja, 2010). Plants have developed intricate defense mechanisms to cope with excess ROS, which include an antioxidant enzyme system. Notably, several enzymes, including CAT, POD, and SOD, played significant roles in the antioxidant enzyme system (Gill and Tuteja, 2010). Relative to wild type, the contents of MDA and hydrogen peroxide in overexpression lines were significantly decreased when exposed to salt and drought stresses, suggesting that there is lower oxidative damage in overexpression lines (Figure 8 and Supplementary Figure 7). Furthermore, the enzyme activities of CAT, POD, and SOD in overexpression lines were significantly higher than those of wild type under salt and drought stresses, suggesting that overexpression lines increased the capacity of the ROS-scavenging system (Figure 8). It was reported that NAC transcription factors usually participate in response to abiotic stresses through regulating the expression of downstream stress-responsive genes (He et al., 2019b). AtNAP functions to negatively regulate salt stress tolerance through repressing *ARBE1* (Seok et al., 2017). In this study, we found that *NtNAC053* encoded a nuclear localization protein and possessed transcriptional activation ability (Figures 2, 3). Many aquaporins genes were reported to be responsive to abiotic stress during different developing stages. In tobacco, over-expression of the *NtAQP1* gene improves water use efficiency, hydraulic conductivity, and yield production under abiotic stress (Sade et al., 2010). Notably, under drought stress treatments, the drought stress-responsive gene *NtAQP1* was significantly higher in the *NtNAC053* overexpression lines (Supplementary Figure 6). Furthermore, NtNAC053 could significantly activate several stress-responsive genes, including *NtCOR15A, NtRAB18, NtDREB1A, NtERF5, NtKAT2*, and *NtERD11* (Figure 9). Besides, several ion channels encoding genes, including *NHX1* and *SOS1*, have been reported to be involved in the ion homeostasis and salt stress responses. These genes were significantly higher in overexpression lines (Supplementary Figure 8). In addition, promoter analysis revealed that all these stress-responsive genes promoters possessed NAC-binding sites (Supplementary Table 2). Yeast one-hybrid assay implied that NtNAC053 might be combined with NACRS (Figure 10), suggesting NtNAC053 could activate these stress-responsive genes by binding their promoter directly.

## Conclusion

In this study, a typical *NAC* gene *NtNAC053* was cloned from tobacco K326 and functionally verified in overexpressing tobacco. *NtNAC053* overexpression lines showed significantly increased resistance to salt and drought stresses, while the overexpression lines seemed to be more sensitive to ABA treatment. Taken together, the transcription factor NtNAC053 might be involved in salt and drought stress responses *via* ABA-mediated signaling in tobacco.

## Data Availability Statement

The original contributions presented in the study are included in the article/Supplementary Material, further inquiries can be directed to the corresponding author/s.

## Author Contributions

XL, QW, and CG conducted the research and participated in drafting the manuscript. JS, ZL, YW, AY, WP, and YG assisted in data collection and analysis. JG and LW conceived this research, designed the experiments, and drafted the manuscript. All authors contributed to the article and approved the submitted version.

## Conflict of Interest

XL, YW, WP, and JG were employed by China Tobacco Hunan Industrial Co., Ltd. The remaining authors declare that the research was conducted in the absence of any commercial or financial relationships that could be construed as a potential conflict of interest.

## Publisher’s Note

All claims expressed in this article are solely those of the authors and do not necessarily represent those of their affiliated organizations, or those of the publisher, the editors and the reviewers. Any product that may be evaluated in this article, or claim that may be made by its manufacturer, is not guaranteed or endorsed by the publisher.

## Footnotes

1
http://solgenomics.net/

2
https://web.expasy.org/protparam/

3
https://www.novopro.cn/tools/nls-signal-prediction.html

4
https://www.arabidopsis.org/

5
http://meme-suite.org/

6
http://bioinformatics.psb.ugent.be/webtools/plantcare/html/

## Supplementary Material

Supplementary Figure 1 Multiple sequence alignment of NtNAC053 with reported NAC proteins from *Arabidopsis* and potato, including ANAC002, ANAC081, ANAC029, ANAC072, ANAC055, ANAC019, and StNAC053. The black lines indicated five subdomains **(A–E)** and nuclear location signal (NLS).

Supplementary Figure 2 The phylogenetic, syntenic analysis and motif organization of NtNAC053. **(A)** The phylogenetic tree was generated based on the amino acid sequences of NtNAC053 and other reported NAC proteins. **(B)** The motif organizations were predicted by MEME. **(C)** The gray line in the background represents the collinear blocks between tobacco and potato, while the red line exhibits the syntenic NAC gene pairs.

Supplementary Figure 3 The putative conserved motifs in NAC proteins.

Supplementary Figure 4 The *cis*-acting element analysis of the promoter regions of *NtNAC053* gene.

Supplementary Figure 5 The expression level of the *NtNAC053* gene in wild type and two overexpression lines, the ratios of gene expression levels were calculated relative to the wild type.

Supplementary Figure 6 Expression of *NtAQP1* in overexpression lines and wild-type tobacco under drought stress. The ratios of gene expression levels were calculated relative to the control, respectively.

Supplementary Figure 7 Analysis of H_2_O_2_ contents in overexpression lines and wild-type tobacco under salt and drought stresses. H_2_O_2_, hydrogen peroxide. WT, wild type.

Supplementary Figure 8 Expression of *NtNHX1* and *NtSOS1* in overexpression lines and wild-type tobacco under salt stress. The ratios of gene expression levels were calculated relative to the control, respectively. WT, wild type.

Supplementary Figure 9 The expressions of ABA signaling genes in overexpression lines and wild-type tobacco under salt and drought stresses. The ratios of gene expression levels were calculated relative to the control, respectively. WT, wild type.

## References

[B1] Aida M. Ishida T. Fukaki H. Fujisawa H. Tasaka M. (1997). Genes involved in organ separation in *Arabidopsis*: an analysis of the cup-shaped cotyledon mutant. *Plant Cell* 9 841–857. 10.1105/tpc.9.6.841 9212461 PMC156962

[B2] Buschmann H. (2016). Plant cell division analyzed by transient Agrobacterium-mediated transformation of tobacco bY-2 cells. *Methods Mol. Biol.* 1370 17–25. 10.1007/978-1-4939-3142-2_2 26659951

[B3] Chen C. Chen H. Zhang Y. Thomas H. R. Frank M. H. He Y. (2020). TBtools: an integrative toolkit developed for interactive analyses of big biological data. *Mol. Plant.* 13 1194–1202. 10.1016/j.molp.2020.06.009 32585190

[B4] Ernst H. A. Olsen A. N. Larsen S. Lo Leggio L. (2004). Structure of the conserved domain of ANAC, a member of the NAC family of transcription factors. *EMBO Rep.* 5 297–303. 10.1038/sj.embor.7400093 15083810 PMC1299004

[B5] Fujita M. Fujita Y. Maruyama K. Seki M. Hiratsu K. Ohme-Takagi M. (2004). A dehydration-induced NAC protein, RD26, is involved in a novel ABA-dependent stress-signaling pathway. *Plant J.* 39 863–876. 10.1111/j.1365-313X.2004.02171.x 15341629

[B6] Gill S. S. Tuteja N. (2010). Reactive oxygen species and antioxidant machinery in abiotic stress tolerance in crop plants. *Plant Physiol. Biochem.* 48 909–930. 10.1016/j.plaphy.2010.08.016 20870416

[B7] Gong X. Zhao L. Song X. Lin Z. Gu B. Yan J. (2019). Genome-wide analyses and expression patterns under abiotic stress of NAC transcription factors in white pear (*Pyrus bretschneideri*). *BMC Plant Biol.* 19:161. 10.1186/s12870-019-1760-8 31023218 PMC6485137

[B8] Guo Y. Gan S. (2006). AtNAP, a NAC family transcription factor, has an important role in leaf senescence. *Plant J.* 46 601–612. 10.1111/j.1365-313X.2006.02723.x 16640597

[B9] He K. Zhao X. Chi X. Wang Y. Jia C. Zhang H. (2019a). A novel Miscanthus NAC transcription factor MlNAC10 enhances drought and salinity tolerance in transgenic *Arabidopsis*. *J. Plant Physiol.* 233 84–93. 10.1016/j.jplph.2019.01.001 30623878

[B10] He L. Bian J. Xu J. Yang K. (2019b). Novel maize NAC transcriptional repressor ZmNAC071 confers enhanced sensitivity to ABA and osmotic stress by downregulating stress-responsive genes in transgenic *Arabidopsis*. *J. Agric. Food Chem.* 67 8905–8918. 10.1021/acs.jafc.9b02331 31380641

[B11] He X. J. Mu R. L. Cao W. H. Zhang Z. G. Zhang J. S. Chen S. Y. (2005). AtNAC2, a transcription factor downstream of ethylene and auxin signaling pathways, is involved in salt stress response and lateral root development. *Plant J.* 44 903–916. 10.1111/j.1365-313X.2005.02575.x 16359384

[B12] Hendelman A. Stav R. Zemach H. Arazi T. (2013). The tomato NAC transcription factor SlNAM2 is involved in flower-boundary morphogenesis. *J. Exp. Bot.* 64 5497–5507. 10.1093/jxb/ert324 24085581 PMC3871814

[B13] Hibara K. Takada S. Tasaka M. (2003). CUC1 gene activates the expression of SAM-related genes to induce adventitious shoot formation. *Plant J.* 36 687–696. 10.1046/j.1365-313x.2003.01911.x 14617069

[B14] Kim M. J. Park M. J. Seo P. J. Song J. S. Kim H. J. Park C. M. (2012). Controlled nuclear import of the transcription factor NTL6 reveals a cytoplasmic role of SnRK2.8 in the drought-stress response. *Biochem. J.* 448 353–363. 10.1042/BJ20120244 22967043

[B15] Kim S. G. Lee S. Seo P. J. Kim S. K. Kim J. K. Park C. M. (2010). Genome-scale screening and molecular characterization of membrane-bound transcription factors in *Arabidopsis* and rice. *Genomics* 95 56–65. 10.1016/j.ygeno.2009.09.003 19766710

[B16] Larsson E. Sundstrom J. F. Sitbon F. von Arnold S. (2012). Expression of PaNAC01, a picea abies CUP-SHAPED COTYLEDON orthologue, is regulated by polar auxin transport and associated with differentiation of the shoot apical meristem and formation of separated cotyledons. *Ann. Bot.* 110 923–934. 10.1093/aob/mcs151 22778149 PMC3423809

[B17] Li P. Peng Z. Xu P. Tang G. Ma C. Zhu J. (2021). Genome-wide identification of NAC transcription factors and their functional prediction of abiotic stress response in peanut. *Front. Genet.* 12:630292. 10.3389/fgene.2021.630292 33767732 PMC7985091

[B18] Li W. Li X. Chao J. Zhang Z. Wang W. Guo Y. (2018). NAC family transcription factors in tobacco and their potential role in regulating leaf senescence. *Front. Plant Sci.* 9:1900. 10.3389/fpls.2018.01900 30622549 PMC6308388

[B19] Li Z. Chao J. Li X. Li G. Song D. Guo Y. (2021). Systematic analysis of the bZIP family in tobacco and functional characterization of NtbZIP62 involvement in salt stress. *Agronomy* 11:148. 10.3390/agronomy11010148

[B20] Livak K. J. Schmittgen T. D. (2001). Analysis of relative gene expression data using real-time quantitative PCR and the 2(-Delta Delta C(T)) Method. *Methods* 25 402–408. 10.1006/meth.2001.1262 11846609

[B21] Mitsuda N. Iwase A. Yamamoto H. Yoshida M. Seki M. Shinozaki K. (2007). NAC transcription factors, NST1 and NST3, are key regulators of the formation of secondary walls in woody tissues of *Arabidopsis*. *Plant Cell* 19 270–280. 10.1105/tpc.106.047043 17237351 PMC1820955

[B22] Moore K. Roberts L. J. (1998). Measurement of lipid peroxidation. *Free Radic. Res.* 28 659–671. 10.3109/10715769809065821 9736317

[B23] Nakashima K. Takasaki H. Mizoi J. Shinozaki K. Yamaguchi-Shinozaki K. (2012). NAC transcription factors in plant abiotic stress responses. *Biochim. Biophys. Acta* 1819 97–103. 10.1016/j.bbagrm.2011.10.005 22037288

[B24] Nuruzzaman M. Manimekalai R. Sharoni A. M. Satoh K. Kondoh H. Ooka H. (2010). Genome-wide analysis of NAC transcription factor family in rice. *Gene* 465 30–44. 10.1016/j.gene.2010.06.008 20600702

[B25] Olsen A. N. Ernst H. A. Leggio L. L. Skriver K. (2005). NAC transcription factors: structurally distinct, functionally diverse. *Trends Plant Sci.* 10 79–87. 10.1016/j.tplants.2004.12.010 15708345

[B26] Ooka H. Satoh K. Doi K. Nagata T. Otomo Y. Murakami K. (2003). Comprehensive analysis of NAC family genes in *Oryza sativa* and *Arabidopsis thaliana*. *DNA Res.* 10 239–247. 10.1093/dnares/10.6.239 15029955

[B27] Park J. Kim Y. S. Kim S. G. Jung J. H. Woo J. C. Park C. M. (2011). Integration of auxin and salt signals by the NAC transcription factor NTM2 during seed germination in *Arabidopsis*. *Plant Physiol.* 156 537–549. 10.1104/pp.111.177071 21450938 PMC3177257

[B28] Puranik S. Bahadur R. P. Srivastava P. S. Prasad M. (2011). Molecular cloning and characterization of a membrane associated NAC family gene, SiNAC from foxtail millet [Setaria italica (L.) P. Beauv]. *Mol. Biotechnol.* 49 138–150. 10.1007/s12033-011-9385-7 21312005

[B29] Qu L. J. Zhu Y. X. (2006). Transcription factor families in *Arabidopsis*: major progress and outstanding issues for future research. *Curr. Opin. Plant Biol.* 9 544–549. 10.1016/j.pbi.2006.07.005 16877030

[B30] Sade N. Gebretsadik M. Seligmann R. Schwartz A. Wallach R. Moshelion M. (2010). The role of tobacco Aquaporin1 in improving water use efficiency, hydraulic conductivity, and yield production under salt stress. *Plant Physiol.* 152 245–254. 10.1104/pp.109.145854 19939947 PMC2799360

[B31] Seok H. Y. Woo D. H. Nguyen L. V. Tran H. T. Tarte V. N. Mehdi S. M. (2017). Arabidopsis AtNAP functions as a negative regulator via repression of AREB1 in salt stress response. *Planta* 245 329–341. 10.1007/s00425-016-2609-0 27770200

[B32] Shao H. Wang H. Tang X. (2015). NAC transcription factors in plant multiple abiotic stress responses: progress and prospects. *Front. Plant Sci.* 6:902. 10.3389/fpls.2015.00902 26579152 PMC4625045

[B33] Shiriga K. Sharma R. Kumar K. Yadav S. K. Hossain F. Thirunavukkarasu N. (2014). Genome-wide identification and expression pattern of drought-responsive members of the NAC family in maize. *Meta. Gene* 2 407–417. 10.1016/j.mgene.2014.05.001 25606426 PMC4287890

[B34] Singh A. K. Sharma V. Pal A. K. Acharya V. Ahuja P. S. (2013). Genome-wide organization and expression profiling of the NAC transcription factor family in potato (*Solanum tuberosum* L.). *DNA Res.* 20 403–423. 10.1093/dnares/dst019 23649897 PMC3738166

[B35] Souer E. van Houwelingen A. Kloos D. Mol J. Koes R. (1996). The no apical meristem gene of petunia is required for pattern formation in embryos and flowers and is expressed at meristem and primordia boundaries. *Cell* 85 159–170. 10.1016/s0092-8674(00)81093-4 8612269

[B36] Takada S. Hibara K. Ishida T. Tasaka M. (2001). The CUP-SHAPED COTYLEDON1 gene of *Arabidopsis* regulates shoot apical meristem formation. *Development* 128 1127–1135. 10.1007/s004290100164 11245578

[B37] Takasaki H. Maruyama K. Kidokoro S. Ito Y. Fujita Y. Shinozaki K. (2010). The abiotic stress-responsive NAC-type transcription factor OsNAC5 regulates stress-inducible genes and stress tolerance in rice. *Mol. Genet. Genomics* 284 173–183. 10.1007/s00438-010-0557-0 20632034

[B38] Tang Y. Liu M. Gao S. Zhang Z. Zhao X. Zhao C. (2012). Molecular characterization of novel TaNAC genes in wheat and overexpression of TaNAC2a confers drought tolerance in tobacco. *Physiol. Plant* 144 210–224. 10.1111/j.1399-3054.2011.01539.x 22082019

[B39] Tran L. S. Nakashima K. Sakuma Y. Simpson S. D. Fujita Y. Maruyama K. (2004). Isolation and functional analysis of *Arabidopsis* stress-inducible NAC transcription factors that bind to a drought-responsive cis-element in the early responsive to dehydration stress 1 promoter. *Plant Cell* 16 2481–2498. 10.1105/tpc.104.022699 15319476 PMC520947

[B40] Wang Q. Guo C. Li Z. Sun J. Deng Z. Wen L. (2021). Potato NAC transcription factor StNAC053 enhances salt and drought tolerance in transgenic *Arabidopsis*. *Int. J. Mol. Sci.* 22:2568. 10.3390/ijms22052568 33806406 PMC7961516

[B41] Wu Y. Deng Z. Lai J. Zhang Y. Yang C. Yin B. (2009). Dual function of *Arabidopsis* ATAF1 in abiotic and biotic stress responses. *Cell Res.* 19 1279–1290. 10.1038/cr.2009.108 19752887

[B42] Xie Q. Frugis G. Colgan D. Chua N. H. (2000). Arabidopsis NAC1 transduces auxin signal downstream of TIR1 to promote lateral root development. *Genes Dev.* 14 3024–3036. 10.1101/gad.852200 11114891 PMC317103

[B43] Yamaguchi M. Ohtani M. Mitsuda N. Kubo M. Ohme-Takagi M. Fukuda H. (2010). VND-INTERACTING2, a NAC domain transcription factor, negatively regulates xylem vessel formation in *Arabidopsis*. *Plant Cell* 22 1249–1263. 10.1105/tpc.108.064048 20388856 PMC2879754

[B44] Yoo S. Y. Kim Y. Kim S. Y. Lee J. S. Ahn J. H. (2007). Control of flowering time and cold response by a NAC-domain protein in *Arabidopsis*. *PLoS One* 2:e642. 10.1371/journal.pone.0000642 17653269 PMC1920552

[B45] Yuan X. Wang H. Cai J. Bi Y. Li D. Song F. (2019). Rice NAC transcription factor ONAC066 functions as a positive regulator of drought and oxidative stress response. *BMC Plant Biol.* 19:278. 10.1186/s12870-019-1883-y 31238869 PMC6593515

[B46] Zhang L. Yao L. Zhang N. Yang J. Zhu X. Tang X. (2018). Lateral root development in potato is mediated by stu-mi164 regulation of NAC transcription factor. *Front. Plant Sci.* 9:383. 10.3389/fpls.2018.00383 29651294 PMC5884874

[B47] Zhang L. Zhang L. Xia C. Zhao G. Jia J. Kong X. (2015). The novel wheat transcription factor TaNAC47 enhances multiple abiotic stress tolerances in transgenic plants. *Front. Plant Sci.* 6:1174. 10.3389/fpls.2015.01174 26834757 PMC4716647

[B48] Zhao J. Liu J. S. Meng F. N. Zhang Z. Z. Long H. Lin W. H. (2016). ANAC005 is a membrane-associated transcription factor and regulates vascular development in *Arabidopsis*. *J. Integr. Plant Biol.* 58 442–451. 10.1111/jipb.12379 26178734 PMC5054944

[B49] Zhao Q. Gallego-Giraldo L. Wang H. Zeng Y. Ding S. Y. Chen F. (2010). An NAC transcription factor orchestrates multiple features of cell wall development in *Medicago truncatula*. *Plant J.* 63 100–114. 10.1111/j.1365-313X.2010.04223.x 20408998

