# Supplementary material for: NtNAC053, A Novel NAC Transcription Factor, Confers Drought and Salt Tolerances in Tobacco
Source: Front Plant Sci. 2022 May 4;13:817106. doi: 10.3389/fpls.2022.817106 (PMC9115565; doi:10.3389/fpls.2022.817106)
Supplement: Supplementary Figure 1 — Multiple sequence alignment of NtNAC053 with reported NAC proteins from Arabidopsis and potato, including ANAC002, ANAC081, ANAC029, ANAC072, ANAC055, ANAC019, and StNAC053. The black lines indicated five subdomains (A–E) and nuclear location signal (NLS). [file Data_Sheet_1.ZIP › Supplementary materials/Supplementary Figure S1.pdf]

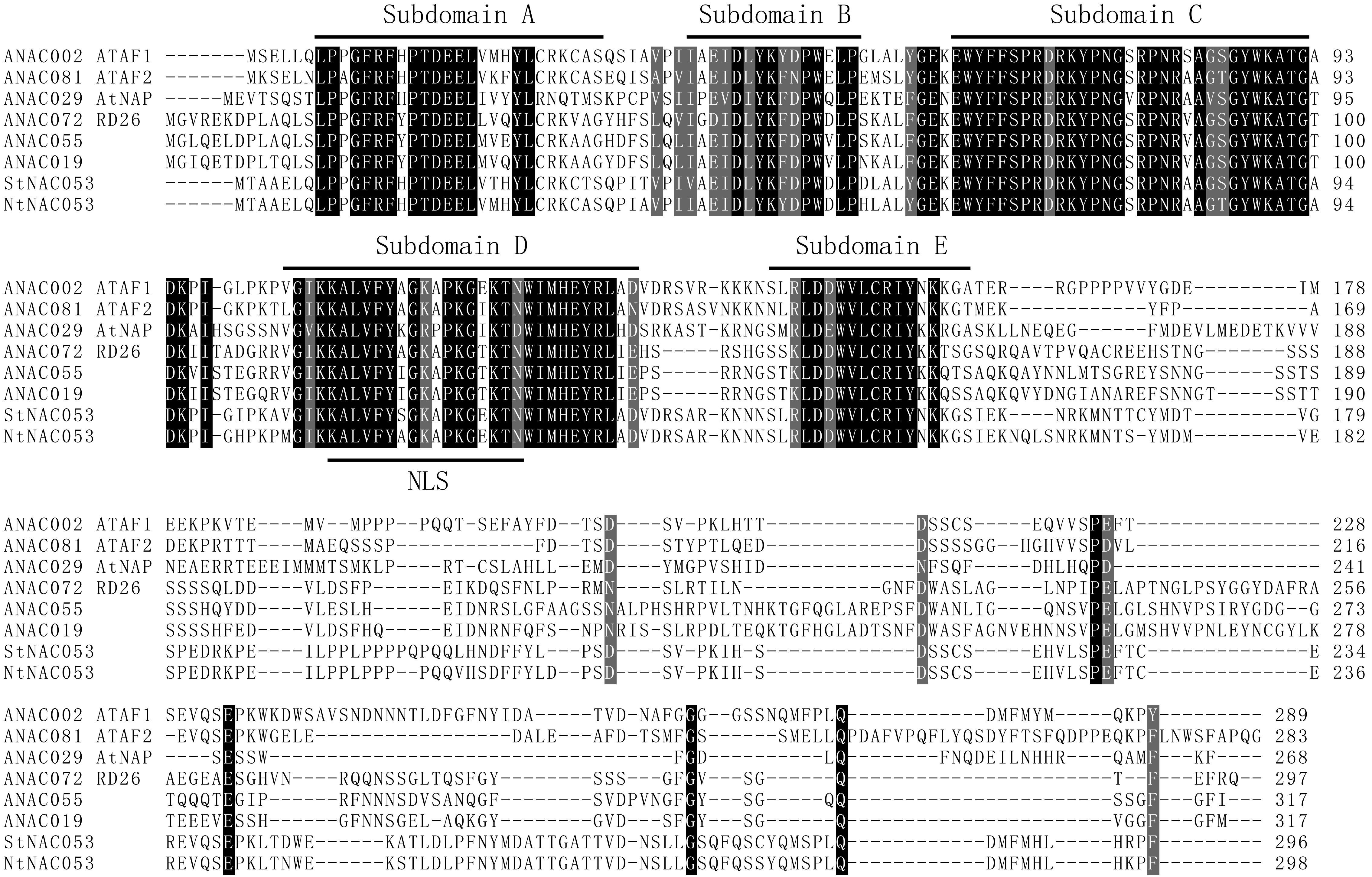
Subdomain A
Subdomain B
Subdomain C
ANAC002 ATAF1 -------MSELLQLPPGFRFHPTDEELVMHYLCRKCASQSIAVPIIAEIDLYKYDPWELPGLALYGEKEWYFFSPRDRKYPNGSRPNRSAGSGYWKATGA 93
ANAC081 ATAF2 -------MKSELNLPAGFRFHPTDEELVKFYLCRKCASEQISAPVIAEIDLYKFNPWELPEMSLYGEKEWYFFSPRDRKYPNGSRPNRAAGTGYWKATGA 93
ANAC029 AtNAP -----MEVTSQSTLPPGFRFHPTDEELIVYYLRNQTMSKPCPVSIIPEVDIYKFDPWQLPEKTEFGENEWYFFSPRERKYPNGVRPNRAAVSGYWKATGT 95
ANAC072 RD26 MGVREKDPLAQLSLPPGFRFYPTDEELLVQYLCRKVAGYHFSLQVIGDIDLYKFDPWDLPSKALFGEKEWYFFSPRDRKYPNGSRPNRVAGSGYWKATGT 100
ANAC055 MGLQELDPLAQLSLPPGFRFYPTDEELMVEYLCRKAAGHDFSLQLIAEIDLYKFDPWVLPSKALFGEKEWYFFSPRDRKYPNGSRPNRVAGSGYWKATGT 100
ANAC019 MGIQETDPLTQLSLPPGFRFYPTDEELMVQYLCRKAAGYDFSLQLIAEIDLYKFDPWVLPNKALFGEKEWYFFSPRDRKYPNGSRPNRVAGSGYWKATGT 100
StNAC053 ------MTAAELQLPPGFRFHPTDEELVTHYLCRKCTSQPITVPIVAEIDLYKFDPWDLPDLALYGEKEWYFFSPRDRKYPNGSRPNRAAGSGYWKATGA 94
NtNAC053 ------MTAAELQLPPGFRFHPTDEELVMHYLCRKCASQPIAVPIIAEIDLYKYDPWDLPHLALYGEKEWYFFSPRDRKYPNGSRPNRAAGTGYWKATGA 94
Subdomain D
Subdomain E
ANAC002 ATAF1 DKPI-GLPKPVGIKKALVFYAGKAPKGEKTNWIMHEYRLADVDRSVR-KKKNSLRLDDWVLCRIYNKKGATER----RGPPPPVVYGDE---------IM 178
ANAC081 ATAF2 DKPI-GKPKTLGIKKALVFYAGKAPKGIKTNWIMHEYRLANVDRSASVNKKNNLRLDDWVLCRIYNKKGTMEK------------YFP-----------A 169
ANAC029 AtNAP DKAIHSGSSNVGVKKALVFYKGRPPKGIKTDWIMHEYRLHDSRKAST-KRNGSMRLDEWVLCRIYKKRGASKLLNEQEG------FMDEVLMEDETKVVV 188
ANAC072 RD26 DKIITADGRRVGIKKALVFYAGKAPKGTKTNWIMHEYRLIEHS-----RSHGSSKLDDWVLCRIYKKTSGSQRQAVTPVQACREEHSTNG-------SSS 188
ANAC055 DKVISTEGRRVGIKKALVFYIGKAPKGTKTNWIMHEYRLIEPS-----RRNGSTKLDDWVLCRIYKKQTSAQKQAYNNLMTSGREYSNNG------SSTS 189
ANAC019 DKIISTEGQRVGIKKALVFYIGKAPKGTKTNWIMHEYRLIEPS-----RRNGSTKLDDWVLCRIYKKQSSAQKQVYDNGIANAREFSNNGT-----SSTT 190
StNAC053 DKPI-GIPKAVGIKKALVFYSGKAPKGEKTNWIMHEYRLADVDRSAR-KNNNSLRLDDWVLCRIYNKKGSIEK----NRKMNTTCYMDT---------VG 179
NtNAC053 DKPI-GHPKPMGIKKALVFYAGKAPKGEKTNWIMHEYRLADVDRSAR-KNNNSLRLDDWVLCRIYNKKGSIEKNQLSNRKMNTS-YMDM---------VE 182
NLS
ANAC002 ATAF1 EEKPKVTE----MV--MPPP--PQQT-SEFAYFD--TSD----SV-PKLHTT-------------DSSCS-----EQVVSPEFT--------------- 228
ANAC081 ATAF2 DEKPRTTT----MAEQSSSP------------FD--TSD----STYPTLQED-------------DSSSSGG--HGHVVSPDVL---------------- 216
ANAC029 AtNAP NEAERRTEEEIMMMTSMKLP----RT-CSLAHLL--EMD----YMGPVSHID-------------NFSQF-----DHLHQPD----------------- 241
ANAC072 RD26 SSSSQLDD----VLDSFP------EIKDQSFNLP--RMN----SLRTILN-----------GNFDWASLAG----LNPIPELAPTNGLPSYGGYDAFRA 256
ANAC055 SSSHQYDD----VLESLH------EIDNRSLGFAAGSSNALPHSHRPVLTNHKTGFQGLAREPSFDWANLIG----QNSVPELGLSHNVPSIRYGDG--G 273
ANAC019 SSSSHFED----VLDSFHQ-----EIDNRNFQFS--NPNRIS-SLRPDLTEQKTGFHGLADTSNFDWASFAGNVEHNNSVPELGMSHVVPNLEYNCGYLK 278
StNAC053 SPEDRKPE----ILPPLPPPPQPQQLHNDFFYL---PSD----SV-PKIH-S-------------DSSCS-----EHVLSPEFTC--------------E 234
NtNAC053 SPEDRKPE----ILPPLPPP--PQQVHSDFFYLD--PSD----SV-PKIH-S-------------DSSCS-----EHVLSPEFTC--------------E 236
ANAC002 ATAF1 SEVQSEPKWKDWSAVSNDNNNTLDFGFNYIDA-----TVD-NAFGG--GSSNQMFPLQ------------DMFMYM-----QKPY--------- 289
ANAC081 ATAF2 -EVQSEPKWGELE-----------------DALE---AFD-TSMFGS------SMELLQPDAFVPQFLYQSDYFTSFQDPPEQKPFLNWSFAPQG 283
ANAC029 AtNAP ----SESSW---------------------------------FGD----------LQ--------FNQDEILNHHR----QAMF---KF---- 268
ANAC072 RD26 AEGEAESGHVN----RQQNSSGLTQSFGY--------SSS---GFGV---SG------Q-------------------------T--F---EFRQ-- 297
ANAC055 TQQQTEGIP------RFNNNSDVSANQGF--------SVDPVNGFGY---SG-----QQ------------------------SSGF---GFI--- 317
ANAC019 TEEEVESSH------GFNNSGEL-AQKGY--------GVD---SFGY---SG------Q------------------------VGGF---GFM--- 317
StNAC053 REVQSEPKLTDWE------KATLDLPFNYMDATTGATTVD-NSLLGSQFQSCYQMSPLQ-------------DMFMHL-----HRPF--------- 296
NtNAC053 REVQSEPKLTNWE------KSTLDLPFNYMDATTGATTVD-NSLLGSQFQSSYQMSPLQ-------------DMFMHL-----HKPF--------- 298
