# Supplementary figures and images for: NtNAC053, A Novel NAC Transcription Factor, Confers Drought and Salt Tolerances in Tobacco
Source: Front Plant Sci. 2022 May 4;13:817106. doi: 10.3389/fpls.2022.817106 (PMC9115565; doi:10.3389/fpls.2022.817106)

A

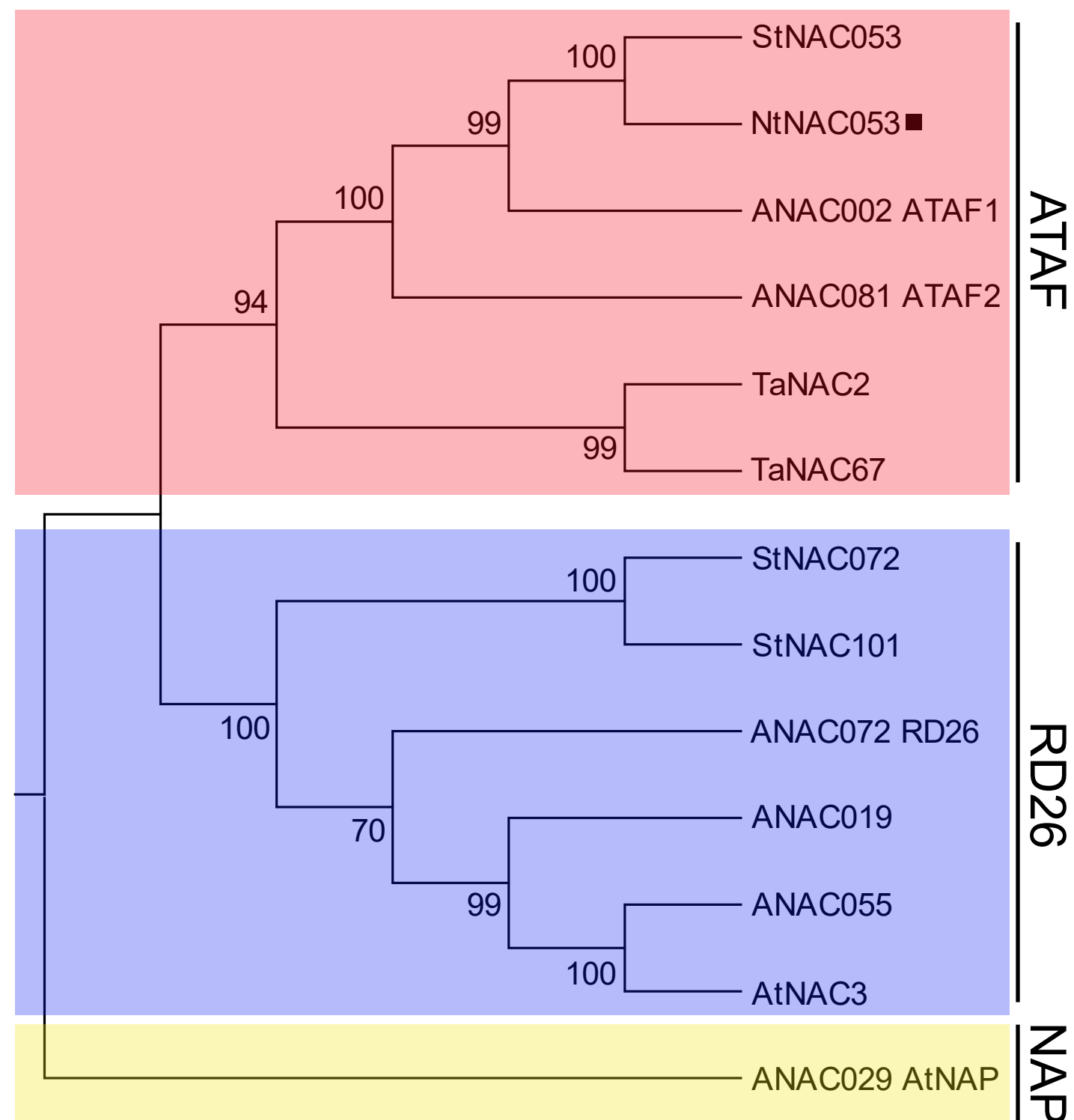

B

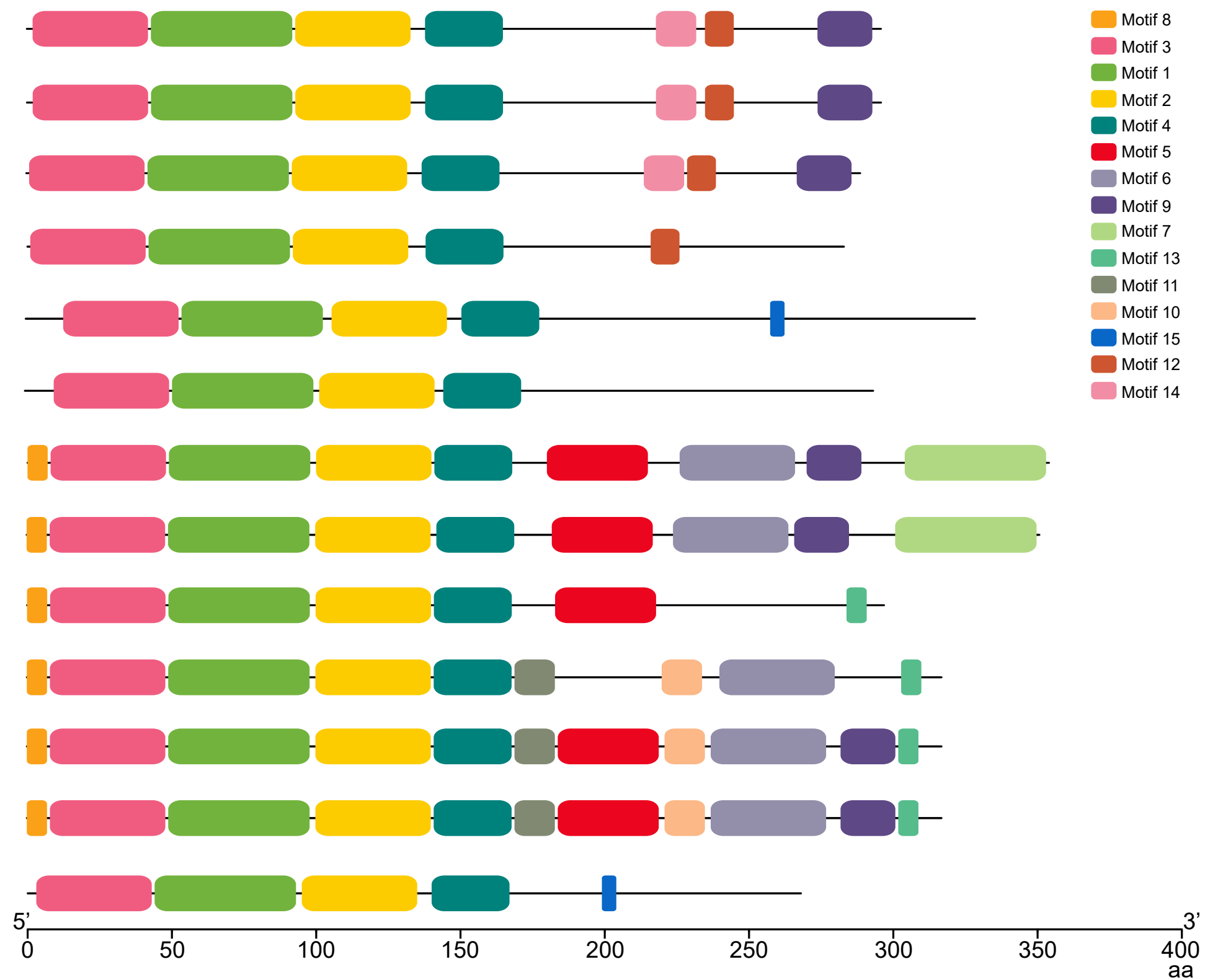

C

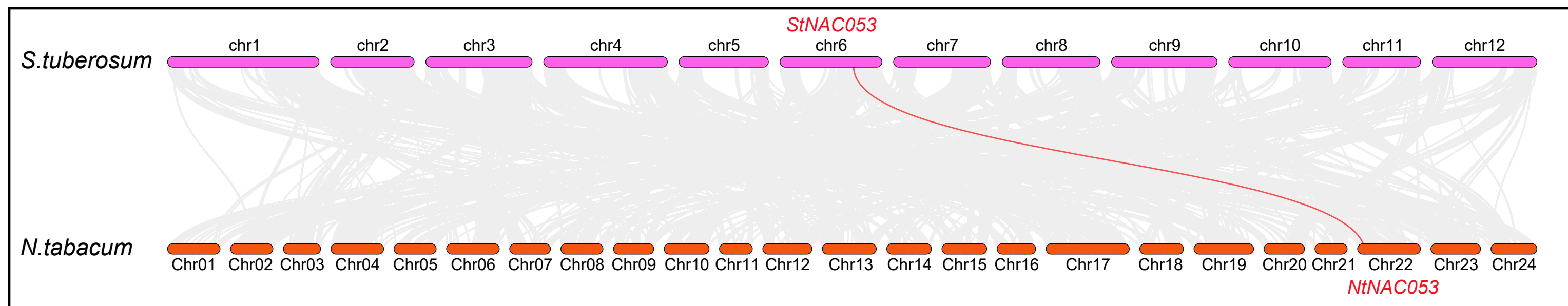

Supplement: Supplementary Figure 1 — Multiple sequence alignment of NtNAC053 with reported NAC proteins from Arabidopsis and potato, including ANAC002, ANAC081, ANAC029, ANAC072, ANAC055, ANAC019, and StNAC053. The black lines indicated five subdomains (A–E) and nuclear location signal (NLS). [file Data_Sheet_1.ZIP › Supplementary materials/Supplementary Figure S2.pdf]

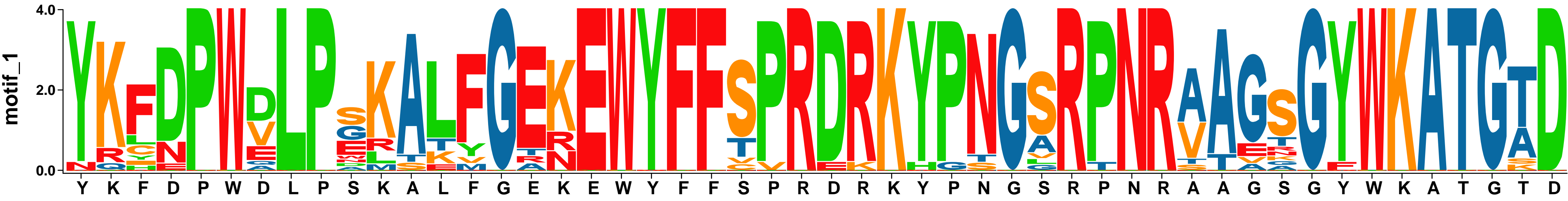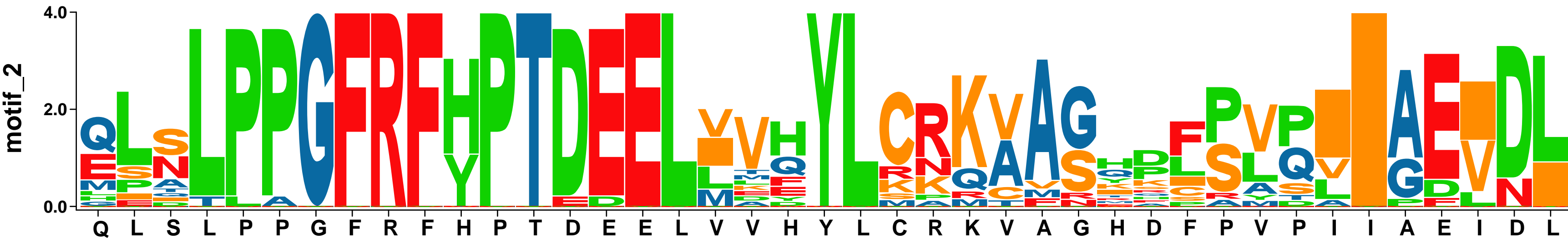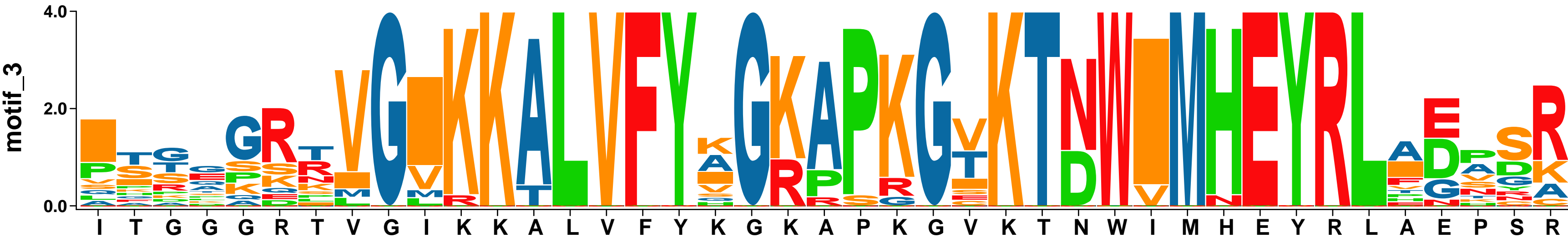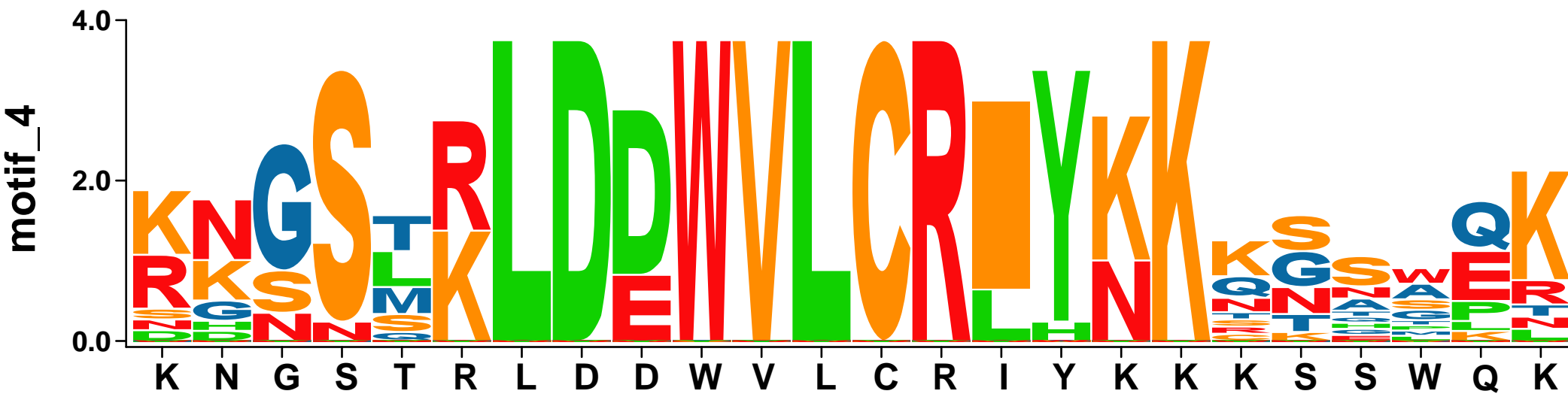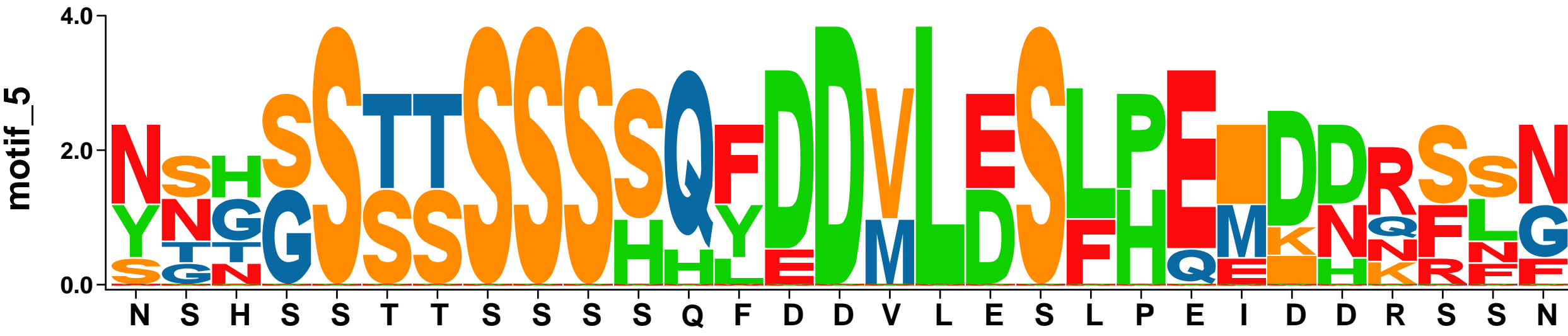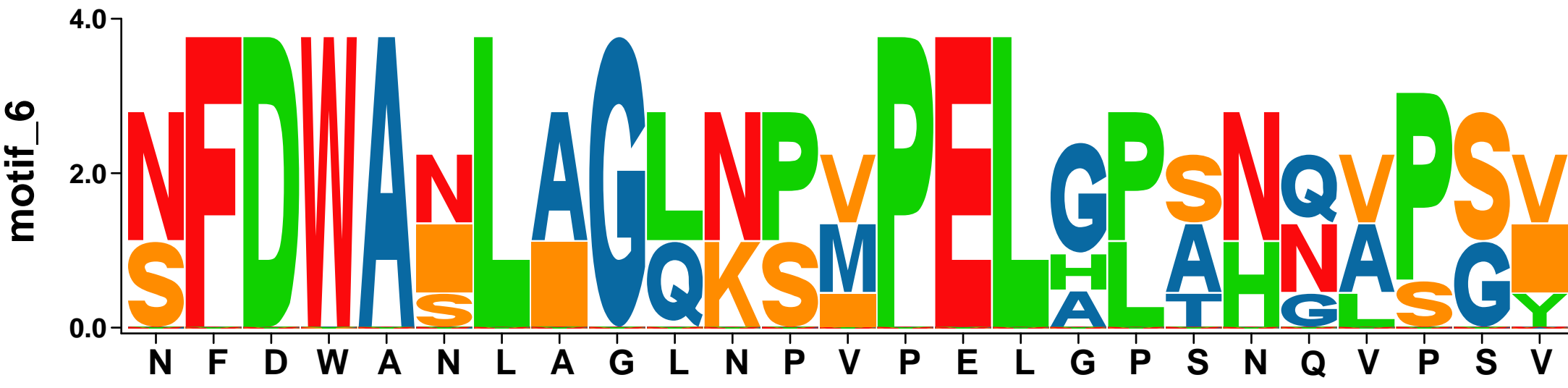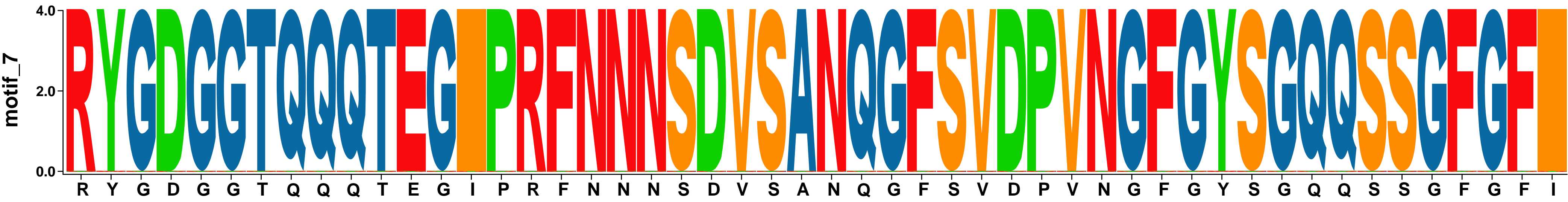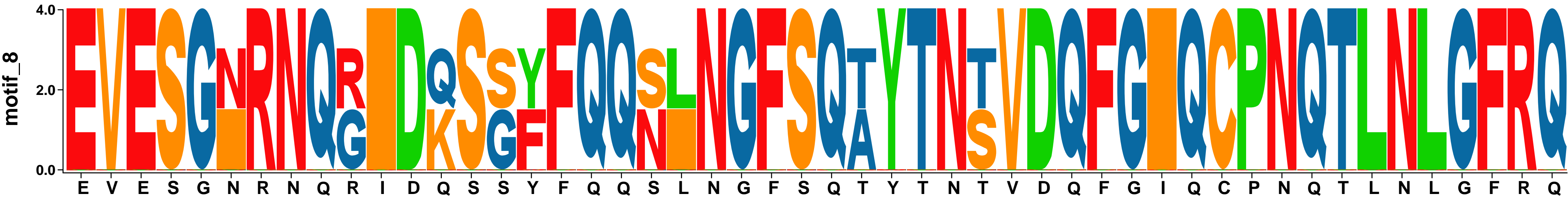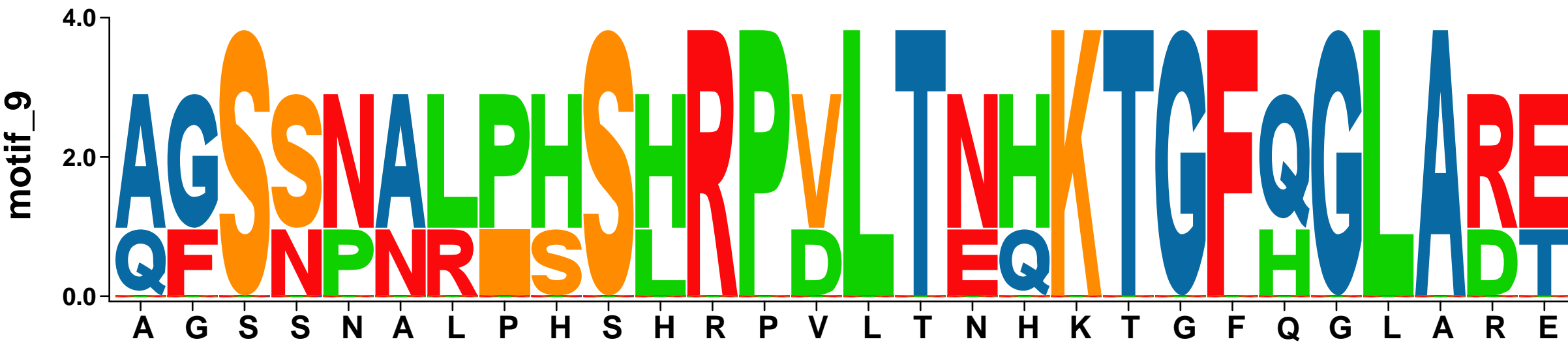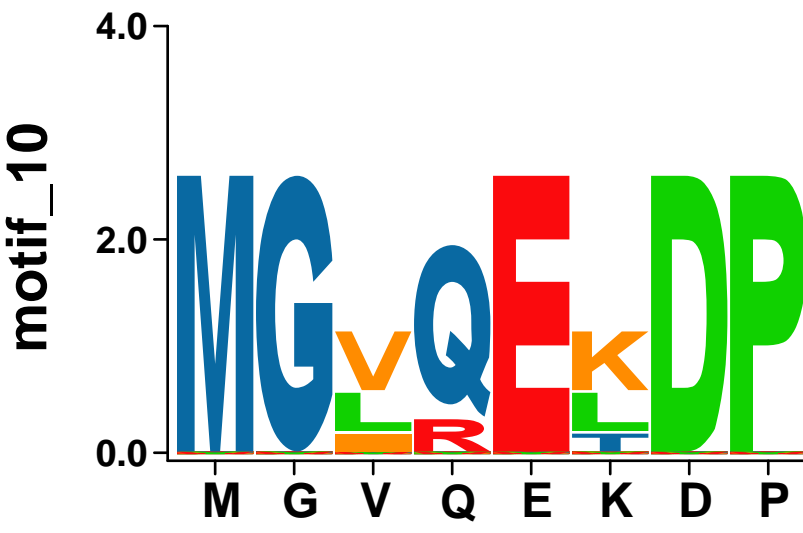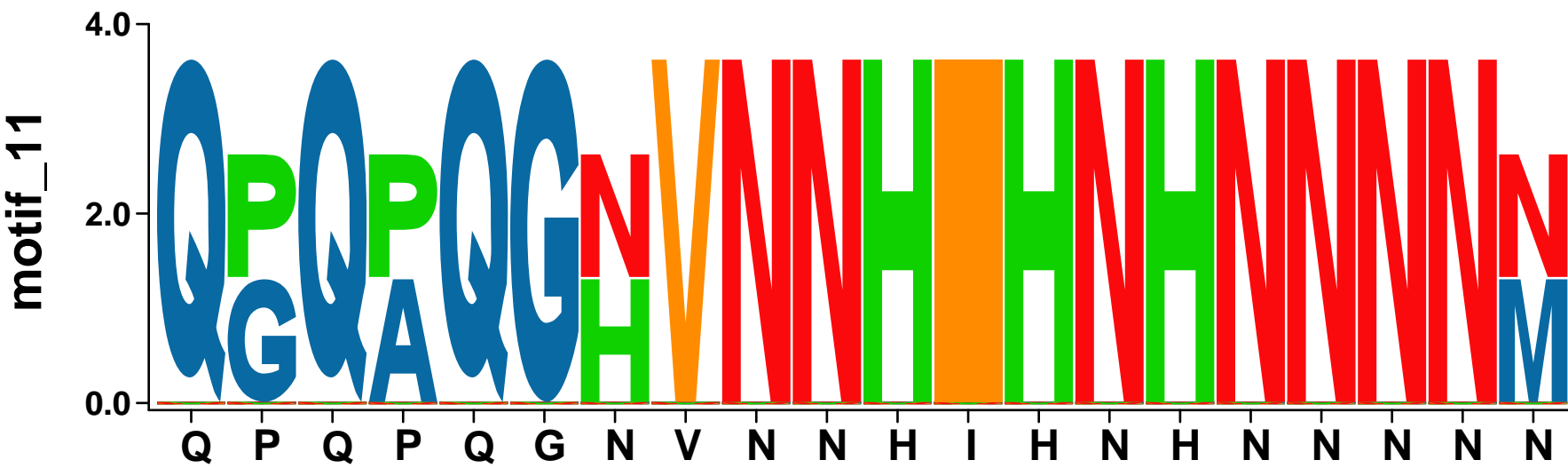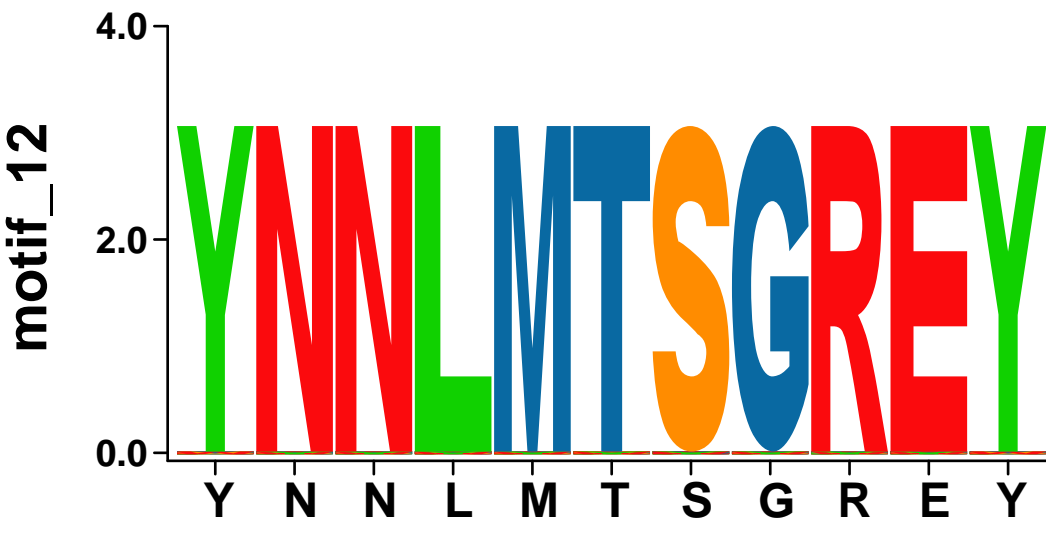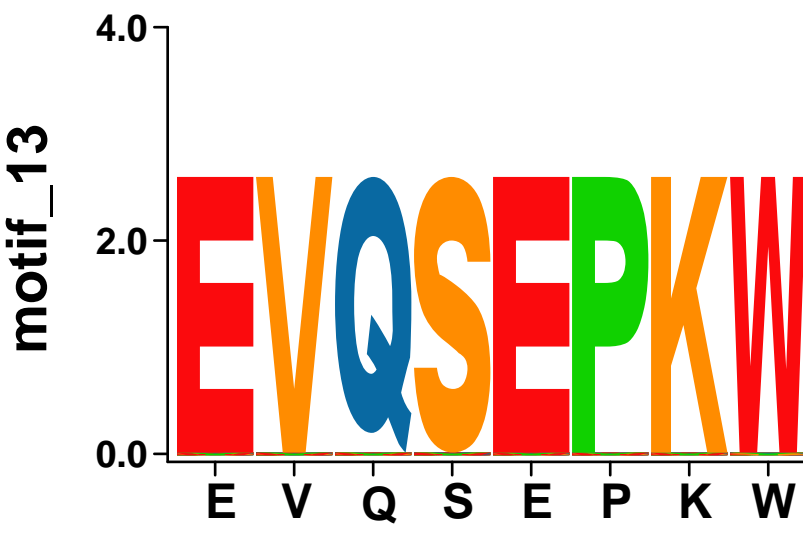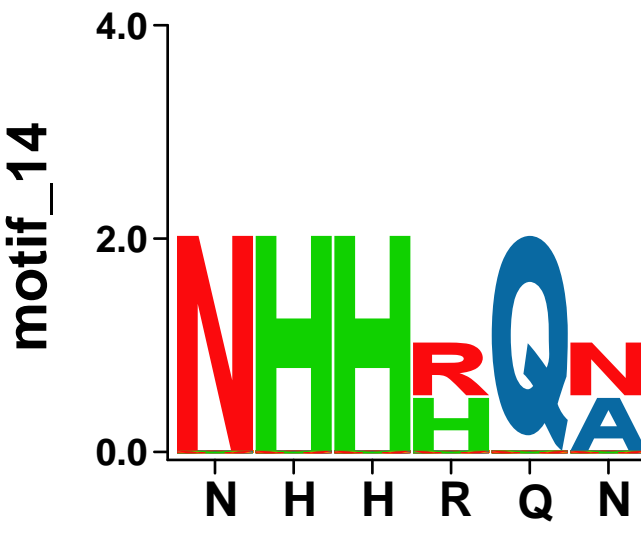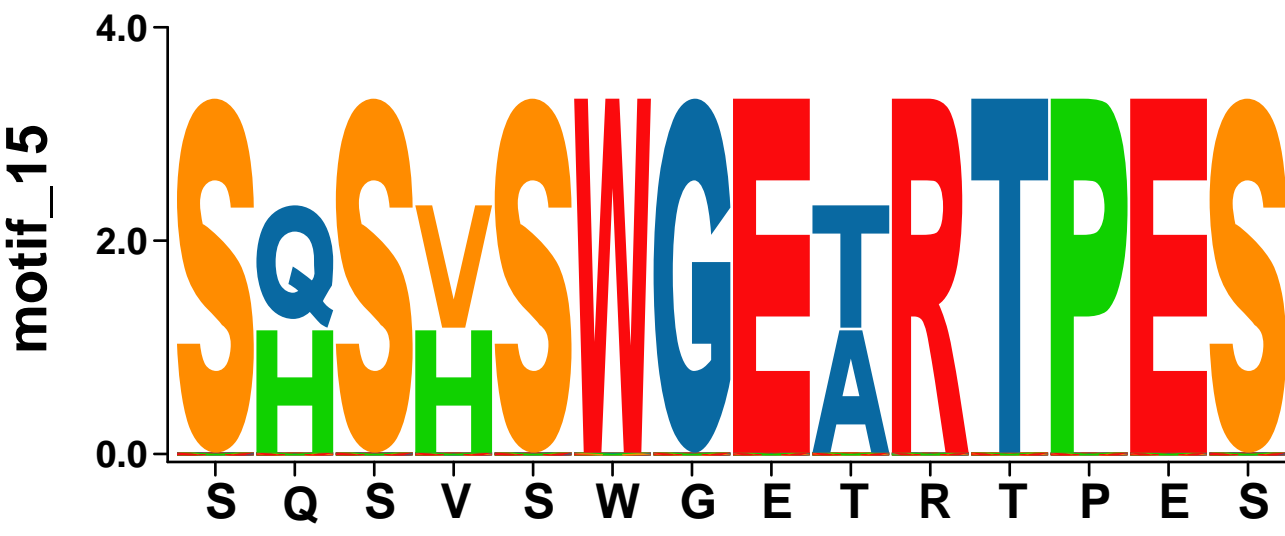

Supplement: Supplementary Figure 1 — Multiple sequence alignment of NtNAC053 with reported NAC proteins from Arabidopsis and potato, including ANAC002, ANAC081, ANAC029, ANAC072, ANAC055, ANAC019, and StNAC053. The black lines indicated five subdomains (A–E) and nuclear location signal (NLS). [file Data_Sheet_1.ZIP › Supplementary materials/Supplementary Figure S3.pdf]

*NtNAC053* Promoter

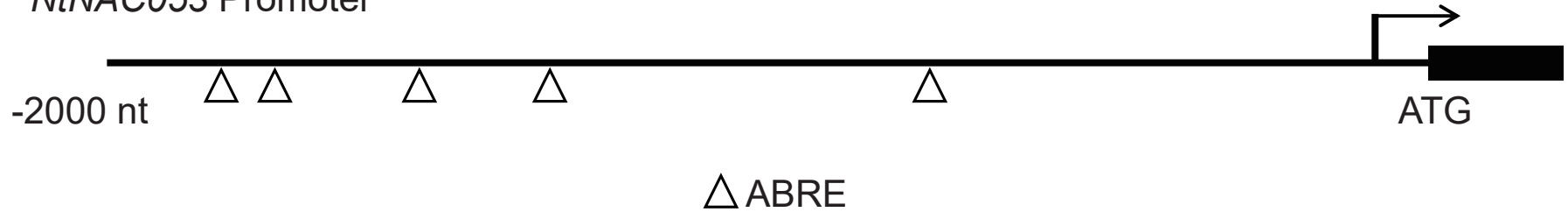

Supplement: Supplementary Figure 1 — Multiple sequence alignment of NtNAC053 with reported NAC proteins from Arabidopsis and potato, including ANAC002, ANAC081, ANAC029, ANAC072, ANAC055, ANAC019, and StNAC053. The black lines indicated five subdomains (A–E) and nuclear location signal (NLS). [file Data_Sheet_1.ZIP › Supplementary materials/Supplementary Figure S4.pdf]

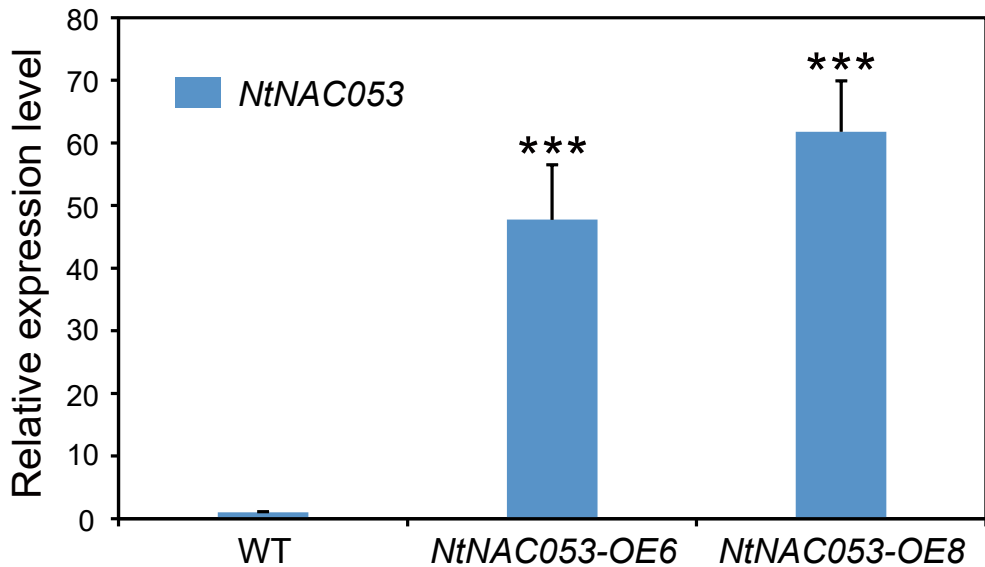

Supplement: Supplementary Figure 1 — Multiple sequence alignment of NtNAC053 with reported NAC proteins from Arabidopsis and potato, including ANAC002, ANAC081, ANAC029, ANAC072, ANAC055, ANAC019, and StNAC053. The black lines indicated five subdomains (A–E) and nuclear location signal (NLS). [file Data_Sheet_1.ZIP › Supplementary materials/Supplementary Figure S5.pdf]

# *NtAQP1*

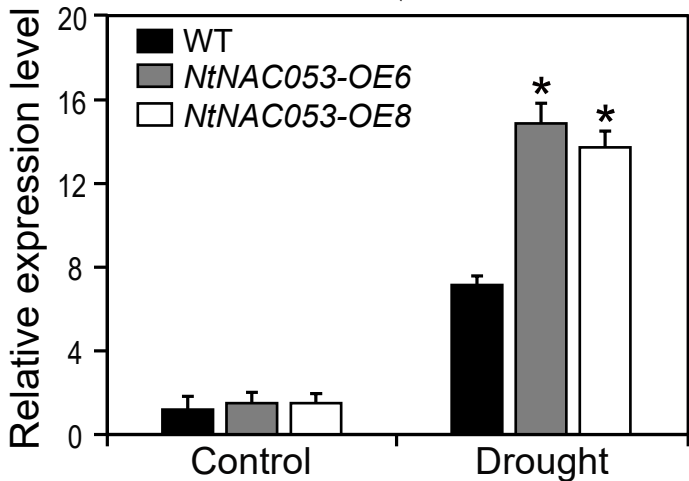

Supplement: Supplementary Figure 1 — Multiple sequence alignment of NtNAC053 with reported NAC proteins from Arabidopsis and potato, including ANAC002, ANAC081, ANAC029, ANAC072, ANAC055, ANAC019, and StNAC053. The black lines indicated five subdomains (A–E) and nuclear location signal (NLS). [file Data_Sheet_1.ZIP › Supplementary materials/Supplementary Figure S6.pdf]

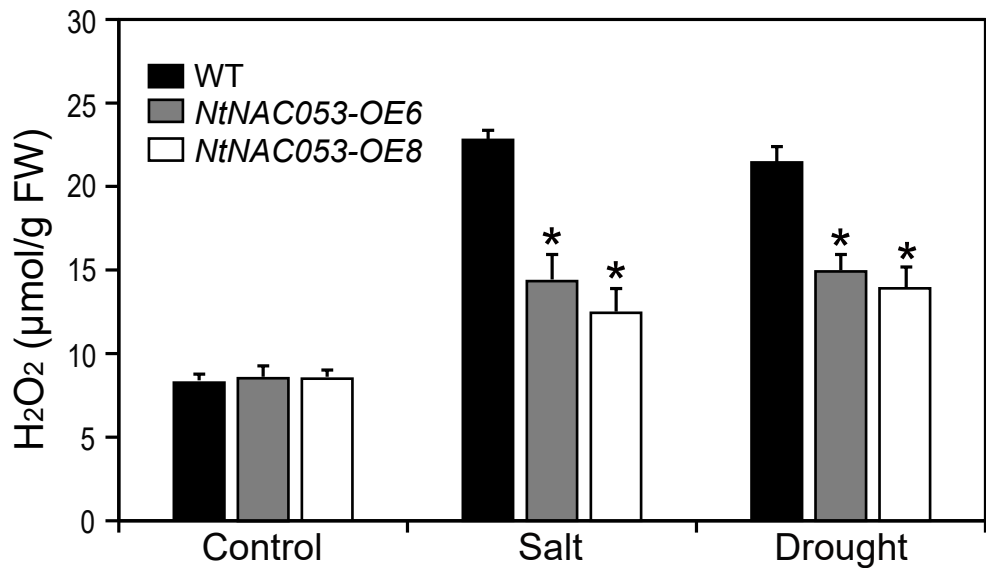

Supplement: Supplementary Figure 1 — Multiple sequence alignment of NtNAC053 with reported NAC proteins from Arabidopsis and potato, including ANAC002, ANAC081, ANAC029, ANAC072, ANAC055, ANAC019, and StNAC053. The black lines indicated five subdomains (A–E) and nuclear location signal (NLS). [file Data_Sheet_1.ZIP › Supplementary materials/Supplementary Figure S7.pdf]

**A***NtNHX1*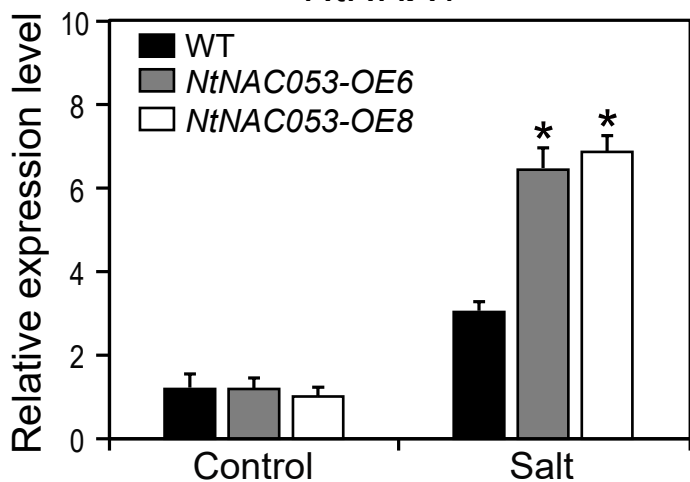**B***NtSOS1*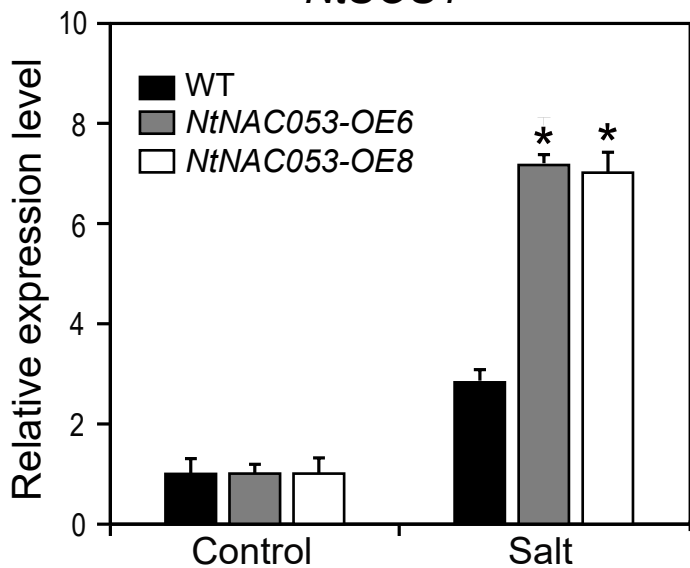

Supplement: Supplementary Figure 1 — Multiple sequence alignment of NtNAC053 with reported NAC proteins from Arabidopsis and potato, including ANAC002, ANAC081, ANAC029, ANAC072, ANAC055, ANAC019, and StNAC053. The black lines indicated five subdomains (A–E) and nuclear location signal (NLS). [file Data_Sheet_1.ZIP › Supplementary materials/Supplementary Figure S8.pdf]

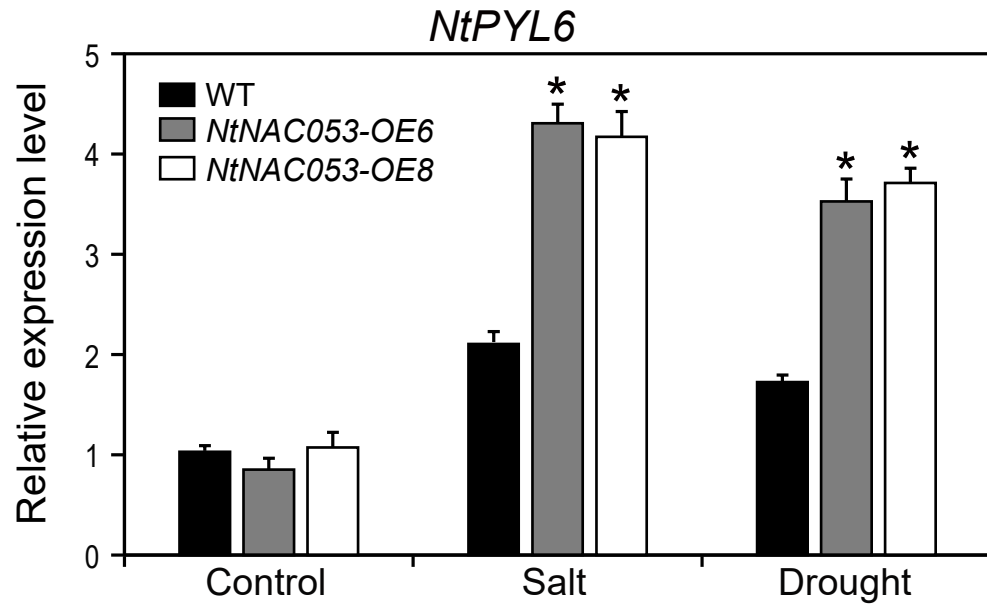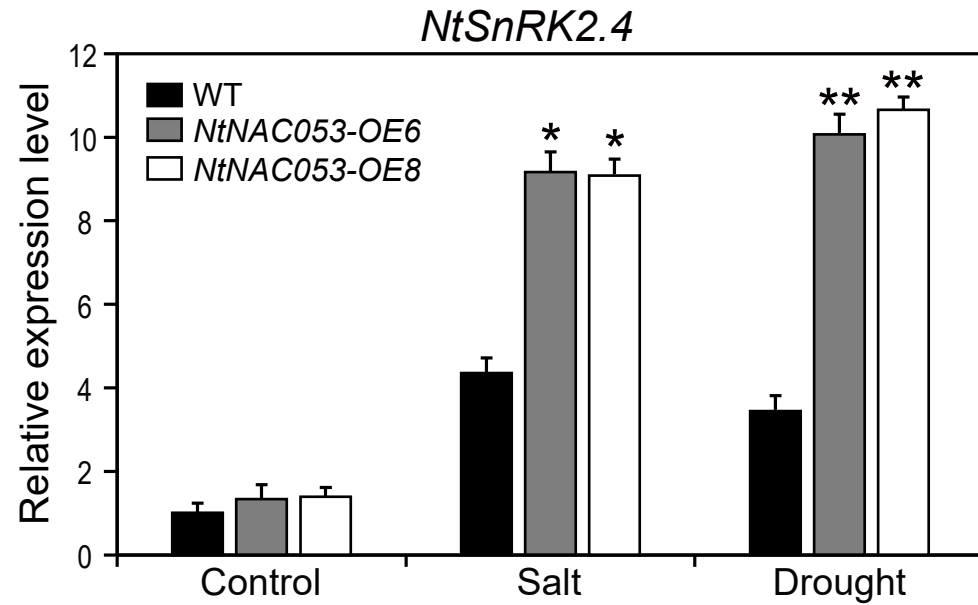

Supplement: Supplementary Figure 1 — Multiple sequence alignment of NtNAC053 with reported NAC proteins from Arabidopsis and potato, including ANAC002, ANAC081, ANAC029, ANAC072, ANAC055, ANAC019, and StNAC053. The black lines indicated five subdomains (A–E) and nuclear location signal (NLS). [file Data_Sheet_1.ZIP › Supplementary materials/Supplementary Figure S9.pdf]
